# Carbonic Anhydrase 3 is required for cardiac repair post myocardial infarction via Smad7-Smad2/3 signaling pathway

**DOI:** 10.7150/ijbs.91396

**Published:** 2024-02-25

**Authors:** Yuanyuan Su, Dongmei Shi, Guofang Xia, Yujia Liu, Longwei Xu, Ling Dao, Xia Lu, Chengxing Shen, Congfeng Xu

**Affiliations:** 1 Department of Cardiology, Shanghai Sixth People's Hospital Affiliated to Shanghai Jiao Tong University School of Medicine, Shanghai 200233, China.; 2 Department of Neurology, Tongren Hospital Affiliated to Shanghai Jiao Tong University School of Medicine, Shanghai 200042, China.; 3 Department of Cardiology, the First Affiliated Hospital of Zhengzhou University, Zhengzhou 450000, China.; 4 Shanghai Key Laboratory of Sleep Disordered Breathing, Shanghai 200233, China.

**Keywords:** cardiac repair, myocardial infarction, fibroblast activation, CAR3, Smad7 acetylation

## Abstract

Appropriate fibrosis is required to prevent subsequent adverse remodeling and heart failure post myocardial infarction (MI), and cardiac fibroblasts (CFs) play a critical role during the process. Carbonic anhydrase 3 (CAR3) is an important mediator in multiple biological processes besides its CO_2_ hydration activity; however, the role and underlying mechanism of CAR3 on cardiac repair post MI injury remains unknown. Here, we found that CAR3 expression was up-regulated in cardiac tissue in infarct area at the reparative phase of MI, with a peak at 7 days post MI. The upregulation was detected mainly on fibroblast instead of cardiomyocyte, and primary cardiac fibroblasts treated with TGF-β1 recaptured our observation. While CAR3 deficiency leads to weakened collagen density, enlarged infarct size and aggravated cardiac dysfunction post-MI. In fibroblast, we observed that CAR3 deficiency restrains collagen synthesis, cell migration and gel contraction of cardiac fibroblasts, whereas overexpression of CAR3 in CFs improves wound healing and cardiac fibroblast activation. Mechanistically, CAR3 stabilizes Smad7 protein via modulating its acetylation, which dampens phosphorylation of Smad2 and Smad3, thus inhibiting fibroblast transformation. In contrast, inhibition of Smad7 acetylation with C646 blunts CAR3 deficiency induced suppression of fibroblast activation and impaired cardiac healing. Our data demonstrate a protective role of CAR3 in cardiac wound repair post MI via promoting fibroblasts activation through Smad7‐TGF-β/Smad2/3 signaling pathway.

## Introduction

Coronary heart disease (CHD) remains the leading cause of death worldwide, of which myocardial infarction (MI) is the largest contributor to cardiovascular morbidity and mortality^1^-^3^. Though substantial improvements have been made in decreasing acute mortality of MI via timely percutaneous coronary intervention over past decades, the risk in survived patients to progressive development of left ventricular (LV) remodeling and heart failure remains unacceptably high^4^-^6^. Cardiac wound healing post-MI undergoes a finely orchestrated series of events including immune cell recruitment and formation of stable infarct scars composed of extracellular matrix (ECM)^5^,^6^. Appropriate healing process is essential for preserving cardiac structure and function, whereas inadequate healing may lead to infarct expansion, severe dilatation of LV, heart failure and even cardiac rupture^5^,^7^,^8^.

During repair processes, timely and well controlled deposition of ECM is critical for stable collagen-based scar formation in the infarct area^9^. And fibroblasts are the dominant cells during the proliferative phase for its crucial role during synthesis and secretion of structural ECM^10^-^12^. In response to stimuli, quiescent fibroblasts can be activated and transformed into myofibroblasts with featured expression of α-smooth muscle actin (⍺-SMA), and enhanced migratory, proliferative, contractile and secretory properties^7^,^11^,^13^. Of all the well-established factors, TGF-β and its downstream signaling have been highlighted to play a central role in myofibroblast differentiation post-MI^12^,^14^-^17^. Considering the complicated and diverse biology of myofibroblast differentiation in early infarct repair and subsequent cardiac remodeling, it is of greatly value to further understand the regulatory factors involved in TGF-β signaling for development of therapeutics.

Carbonic anhydrases (CAs) are a family of zinc-containing metalloenzymes that catalyze the reversible hydration of CO_2_ (CO_2_ + H_2_O ⟷ HCO_3_^-^ + H^+^), allowing these enzymes to regulate intra- and extra-cellular concentrations of CO_2_, and pH value^18^,^19^. Among sixteen different members identified so far, there are eight cytosolic proteins (CA1, CA2, CA3, CA7, CA8, CA10, CA11, CA13), three transmembrane proteins (CA9, CA12, CA14), two mitochondrial matrix proteins (CA5A, CA5B), two glycosylphosphatidylinositol (GPI)-anchored proteins (CA4 and CA15) and one secreted protein (CA6). Extensive evidence has underscored the participation of CAs in numerous biological processes, such as adipogenesis, gluconeogenesis, acid-base balance, ion transport and calcification^20^,^21^. Among the family, carbonic anhydrase 3 (CA3) is special because of its negligent catalytic capability (about only 3 % of that of CA2)^22^,^23^. Mainly expressed in skeletal muscle, liver and adipose tissues, CA3 has been verified to involve in various biological processes including alleviation of oxidative stress^24^-^27^, regulation of lipogenesis^28^-^30^, promotion of cell migration^31^,^32^. Recently, CA3 has been detected in heart tissue^22^, and may involve in multiple cardiovascular diseases including dilated cardiomyopathy (DCM) with HF^33^, hypertrophic cardiomyopathy (HCM)^34^, doxorubicin-induced cardiomyopathy^35^, and ischemia-reperfusion Injury^36^, suggesting a potential role of CA3 in cardiovascular system. However, the exact biological function of CA3 remains unrevealed. In this study, we found that CAR3 (the mouse homolog for human CA3) regulates fibroblast activation through TGF-β/Smad2/3 signaling via mediating acetylation of Smad7. Our study provides evidence that CAR3 mediates fibroblast transformation to promote wound healing post myocardial infarction, and identifies a potential therapeutic mediator for cardiac repair and heart failure post-MI.

## Materials and Methods

### Animals and myocardial infarction model

The animal experimental protocols were reviewed and approved by the Animal Ethics Committee of Shanghai Jiao Tong University. *Car3*‐deficient staggerer (sg/sg) mice were obtained from Jackson Laboratory (Bar Harbor, ME) as previously described^37^. *Car3*‐deficient mice and their wild‐type (WT) littermates aged 8 to 10-week-old were housed at 24°C ± 2°C, humidity of 40% ± 5%, under a constant 12 h light/dark cycle with available water and standard rodent chow *ad libitum*.

The previously described surgical procedures were followed to induce mouse MI model^38^. Briefly, 8-week-old male mice were anesthetized with a mixture of isoflurane (1.5%) and oxygen (0.5 L/min) inhalation and were placed on a temperature-controlled surgical table before the surgical procedure but without ventilation. An incision was performed in the fourth intercostal space of the left chest to expose the heart. The left anterior descending (LAD) coronary artery was permanently ligated 2 mm from the first branch with a 6-0 silk suture after the heart smoothly and gently externalized. Afterwards, the heart is immediately placed back into the intrathoracic space followed by closure of the skin incision. Sham‐operated mice underwent the abovementioned procedure without LAD occlusion. The blind method was used for the operation and subsequent echocardiographic evaluation in this study. Mice were euthanized with isoflurane/oxygen and sacrificed on the corresponding days after MI surgery to obtain their heart samples for corresponding analyses. To inhibit the acetyltransferase activity of p300 *in vivo*, mice were injected intraperitoneally with C646 (15 mg/kg; MCE, USA) or DMSO once a day for consecutive 14 days^39^.

### Echocardiography

*In vivo* cardiac function of mice was evaluated by echocardiography with a high-resolution imaging system (Vevo2100, Visual Sonic Inc., CA) 7 days or 4 weeks after MI, as previously described^40^. The mice were anesthetized using a mixture of isoflurane and oxygen followed by removal of chest hair and placed in the supine position. The two‐dimensional and M-mode echocardiographic views of the mid‐ventricular short axis were acquired at the level of the papillary muscle tips below the mitral valve. Variables including left ventricular internal diameter at the end of diastolic (LVIDd) and systolic (LVIDs) were measured on M‐mode echocardiography. Left ventricular ejection fraction (LVEF), fractional shortening (LVFS) and left ventricular end-diastolic volume (LVEDV) and end-systolic volume (LVESV) were calculated as reported previously^38^,^40^. During echocardiography assessment, the heart rate of mice was maintained between 450 and 550 beats per minute.

### Triphenyltetrazolium chloride (TTC) staining

To assess the infarct size, the TTC assays were performed as previously described in detail^41^. In brief, the heart was harvested and frozen at -80°C, then quickly sliced into five pieces from the apex to base perpendicular to the long axis of the heart. The sections were subsequently incubated in 1% TTC solution (Sigma Aldrich) dissolved in PBS at 37°C for 20 minutes. After stopping the staining process with PBS, the slices were photographed. The infarct size was calculated as the percentage of infarct over the ventricular area. ImageJ software (1.52 V) was used to determine the infarcted area.

### Isolation, culture and treatment of primary cells

Neonatal rat cardiac fibroblasts (NRCFs) and neonatal rat cardiac myocytes (NRCMs) were isolated from 1 to 2-day-old neonatal Sprague-Dawley rats. The hearts were removed from the chest after euthanasia and immediately cut into about 1 mm^3^ cardiac tissues in ice-cold phosphate buffered saline (PBS). The small pieces were then collected into a cell culture dish and digested with 0.25% trypsin (Gibco) at 4°C for 8 hours. After digestion, the supernatants were passed through 70 μm nylon mesh filter, and plated for 2 hours to allow fast-adherent cells (mainly NRCFs) to attach. Afterwards, the medium was replaced with fresh, prewarmed Dulbecco's Modified Eagle Medium (DMEM, Invitrogen Corporation, USA) with 10% fetal bovine serum (FBS) and 1% penicillin/streptomycin. The collected supernatant (mainly NRCMs) was centrifuged and resuspended in DMEM with 10% FBS and BrdU (0.1 mM, inhibiting the proliferation of fibroblasts, Sigma).

Adult mouse primary cardiac fibroblasts (ACFs) were isolated from 8-10 weeks old WT mice or *Car3*-deficient mice. The hearts were minced into small pieces after removing atriums and connective tissues. Subsequently, the pieces were digested with 0.1% type II collagenase (Worthington Biochemical) for 1 hour at 37°C with shaking. Digested cell suspension was resuspended in DMEM with 10% FBS and then passed through 70 μm filter, followed by seeding into culture dish for further experiments.

All kinds of cells were cultured at 37°C in a humidified incubator containing 5% CO_2_. CFs of the 2nd-4th passages cultured in DMEM supplemented with 10% FBS and 1% penicillin/streptomycin were used for subsequent experiments. To induce fibroblast activation, CFs were put on serum‐free DMEM for 24 hours prior to stimulation with 10 ng/ml TGF-β1 (PeproTech) for appropriate time. To inhibit the acetyltransferase activity of P300, CFs were exposed to serum‐free DMEM for 24 hours prior followed by pretreatment with compound C646^42^ (3 μM, MCE, USA) or DMSO followed by the presence or absence of TGF-β1 (10 ng/ml). After purification by differential preplating, NRCMs were seeded at a density of 1 × 10^6^ cells/ml into corresponding culture dishes and cultured with DMEM with 10% FBS. To simulate ischemic injury, the NRCMs were cultured in serum-free and glucose deprived DMEM under hypoxia for 6 hours after starvation with serum‐free DMEM for 12 hours, which was induced by a hypoxia chamber (Thermo, HERA cell 150i) with 5% CO_2_, 1% O_2_, and 94% N_2_. The same protocols were also used for NRCFs/ACFs. Human Umbilical Vein Endothelial Cells (HUVECs) from the Cell Bank of the Chinese Academy of Sciences (Shanghai, China) were cultured in DMEM supplemented with 10% FBS and 1% penicillin/streptomycin.

### Construction and transduction of recombinant adenovirus

Recombinant adenovirus (Adv) packaged by Genechem (Shanghai, China) were used to manipulate the expression of CAR3 in cultured fibroblasts. Gene sequence of *Car3* was referred to Genbank (NM_007606). To overexpress CAR3, CFs were subsequently infected with Adv-GFP (MOI = 30) or Adv-*Car3* (MOI = 30) for 4 hours, and then the medium containing the adenovirus was removed and replaced by DMEM supplemented with 10% FBS. The cells were cultured in serum-free DMEM for 24 hours and subsequently treated with TGF-β1 or not.

### Western blot analysis

Homogenates of cardiac tissue or cultured primary CFs were prepared using RIPA buffer with complete protease and phosphatase inhibitors (Roche). Lysates protein concentrations were quantified using Pierce BCA Protein Assay Kit (Thermo Fisher). Equal amount of protein lysates (20‐40 μg/lane) were used for SDS‐PAGE Gels. The SDS/PAGE Gels proteins were electro‐transferred onto Polyvinylidene difluoride (PVDF) membranes and then incubated overnight at 4°C with primary antibodies. Followed were all the commercially available antibodies used by Western blotting analysis: anti-CAR3 (sc-50715, Santa Cruz, 1:1000), anti-CAR2 (sc-133111, Santa Cruz, 1:1000), anti-α-SMA (ab7817, abcam,1:2000), anti-Collagen1 (72026, CST, 1:1000), anti-Collagen3 (184993, abcam, 1:1000), anti-TGFβR1 (310328, zen-bio, 1:1000), anti-TGFβR2 (346596, zen-bio, 1:1000), anti-Smad7 (sc-365846, Santa Cruz, 1:1000), anti-p-Smad2 (188334, abcam, 1:1000), anti-p-Smad3 (52903, abcam, 1:1000), anti-Smad2/3 (202445, abcam, 1:1000), anti-Ac-lysine (sc-32268, Santa Cruz, 1:1000), anti-P300 (AF6795, Beyotime 1:1000), anti-HSP90 (4877, CST 1:1000), anti-α-tubulin (52866, abcam, 1:1000). α-tubulin and HSP90 served as the loading control. After incubating with the corresponding peroxidase-conjugated secondary antibodies (Jackson ImmunoResearch, 1:20000) for 1 hour at room temperature, the immunoblot bands were detected with the enhanced-chemiluminescent (ECL) system (Tanon, China) and calculated with ImageJ software.

### Real‐time quantitative polymerase chain reaction (RT q‐PCR)

Total RNA was extracted from cardiac tissues or cells using TRIzol Reagent (Sigma-Aldrich, USA), according to the manufacturer's instructions. cDNA was synthesized using HiScript® II Q RT SuperMix for qPCR (+gDNA wiper) (Vazyme, China), and then amplified by AceQ® Universal SYBR qPCR Master Mix (Vazyme, China) in the LightCycler® 96 Real‐Time PCR System (Roche, USA). β-actin was used as the housekeeping gene. Results from LightCycler® 96 analysis software are presented as Ct values and calculated with standard comparative Ct method. The primer sequences utilized for real‐time PCR in this study are presented in Table S1.

### Immunofluorescence and immunohistochemistry staining

For immunofluorescence (IF) staining, paraffin-embedded sections (5-6 μm thick) of heart tissue chips were dewaxed and rehydrated, followed by antigen retrieval in 10 mM citric acid sodium buffer. In addition, cultured cells were washed with PBS and then fixed with 4% paraformaldehyde. The heart sections and fixed cells were subsequently permeabilized with 0.1% Triton X-100 and blocked in PBS containing with 10% goat serum albumin for 1 hour at room temperature. Next, the samples were incubated with indicated primary antibodies overnight at 4°C. The corresponding primary antibodies used were as follows: anti-CAR3 (196835, abcam, 1:100), anti-α-SMA (7817, abcam, 1:100), anti-Vimentin (8978, abcam,1:100), anti-Collagen1 (72026, CST, 1:1000), anti-Collgen3 (184993, abcam, 1:100), anti-cTnT (8295, abcam, 1:100). After washing with PBS, the slices were incubated with appropriate fluorescent-labeled secondary antibodies for 1 hour at room temperature. Nuclei were counterstained with 4′,6-diamidino- 2-phenylindol (DAPI, Invitrogen, USA). Fluorescent images were obtained by fluorescent confocal microscopy (Leica TCS SP8; Leica, Germany) and fluorescent intensity of cells was quantified using ImageJ software.

The myocardial tissue chips were dewaxed and rehydrated for immunohistochemistry. After antigen retrieval with citric acid sodium solution, the washed tissue slices were permeabilized, blocked and stained with primary antibodies against CAR3 (196835, abcam, 1:100; DAPI, Invitrogen) overnight at 4°C. After incubating with the biotin-conjugated secondary antibodies, staining signals were estimated with an Avidin-Biotin Complex peroxidase system. A 3,3′- diaminobenzidine (DAB) peroxidase substrate kit (K5007, DAKO) was used to visualize antibody binding. Bright-field images were obtained by a fluorescence microscope (Leica, Germany).

### Histological analysis

Hearts were harvested from each group under anesthesia and perfused with PBS followed by fixed with 4% paraformaldehyde. After embedding in paraffin, the heart tissue samples were cut into 5μm thick sections and prepared for hematoxylin and eosin (HE) staining to observe general morphology, Masson's trichrome staining and Sirius Red staining to assess collagen content. Infarct size was determined by infarct area/total LV area from each section and expressed as a percentage. In addition, the ventricular wall thickness of the scar was also calculated using ImageJ software.

### Immunoprecipitation/Co-immunoprecipitation

Prepared heart tissue and cell samples were solubilized in immunoprecipitation lysis buffer (20 mM Tris pH7.5, 150 mM NaCl, 1 mM EDTA, 1% Nonidet P-40, 5% glycerol, protease inhibitor cocktail) and precipitated by centrifugation. The prepared tissue and cell supernatants (800 μg) were incubated with anti-CAR3 (Santa Cruz, 1μg per 250 μg of total protein) antibody overnight at 4°C followed by incubation with 30 μL of protein G Agarose beads (Santa Cruz) for 4 hours at 4°C. After being washed three times with cold wash buffer and once with lysis buffer, the immunoprecipitations were resuspended in 30 μL of loading buffer and subjected to western blot for examination of Smad7. Following consecutive washes, the immunoprecipitations containing the CAR3-bound or IgG control-bound proteins were resuspended in 30 μL of loading buffer and subjected to western blot for examination of Smad7. Whole lysate/input sample was used as a control of the immunoprecipitation enrichment. Then, tissue and cell lysates (800 μg) were prepared and carried out by incubation with anti-Smad7 ((Santa Cruz, 1 μg per 250 μg of total protein) antibody overnight at 4°C followed by addition of 30 μL of protein G Agarose beads. The eluted samples were further analyzed via western blotting to detect the Ac-lysine modification of Smad7.

### Cell migration assay

For the wound healing assay, the cardiac fibroblasts isolated from WT or *Car3*-deficient adult mice were plated in 6-well plates. The confluent monolayer cells were then wounded by creating a linear wound area with a 200 μL pipette tip to evaluate the migratory ability of cardiac fibroblasts. The scratched plate was then gently washed to remove detached cells followed by treatment with TGF-β1 or not for 24 hours. Cells were photographed at time of injury and 24 hours after injury. The migratory capacity of fibroblasts was briefly calculated by measuring the wound recovered area using the ImageJ software.

For the transwell assay, a system (Corning Costar, USA) with upper chamber polycarbonate filters of 8 mm pore size was used to carry out migratory assay. ACFs were harvested and seeded at a density of 3 ×10^4^ cells per well to the top chamber in serum-free medium whereas the bottom well was added with DMEM with 10% FBS. After incubation for 24 hours at 37°C, cells on the upper surface of the membrane were wiped off with a cotton swab, the cells on the lower chamber were fixed with 4% paraformaldehyde, followed by staining with 0.01% Crystal Violet (Sigma, USA) and then counted five fields randomly.

### Collagen gel contraction assay

Isolated ACFs were cultured in DMEM with 10% FBS at 37°C in a 5% CO_2_ incubator for further experiments. Digested fibroblasts were suspended in complete media to prepare a mixture with a bovine collagen solution as the kit protocols described (Cell Biolabs, USA) for a final collagen concentration of 1.5 mg/mL collagen and 2 ×10^5^ cells/mL. 250 μL of solution was seeded in a 48-well plate within a suspension cell culture plate and then incubated at 37°C for 1.5 hours for the gel to coagulate. Subsequently, the collagen gel was detached from the edges of the plate with a pipette tip followed by the correspondent stimuli. Pictures of the surface area of each matrix were photographed immediately after release as well as 12, 24, 48, and 72 hours following. Gel area was measured using ImageJ software and normalized to original gel area.

### Statistical analysis

All quantitative values were presented as mean ± SEM. Data distribution normality was determined by Shapiro-Wilk test. When comparing between two groups, the equality of variances was evaluated by the Levene test. Student's t-test (equal variances) or unequal variance t-test (unequal variances) was used for normally distributed variables whereas the Mann-Whitney U test was used for variables not normally distributed. For comparison of more than two groups, the Brown-Forsythe test was used to evaluate the homogeneity of variance. One-way or two-way analysis of variance (ANOVA) followed by the Bonferroni post hoc test was used if equal variances were assumed in multiple‐group comparisons, otherwise the Tamhane post hoc test was selected if equal variances were not assumed. Statistical analysis was carried out using SPSS (version 26.0) or GraphPad Prism (ver. 9). *p* < 0.05 was regarded as statistically significant.

## Results

### Increased expression of CAR3 in cardiac tissues at the infarction site

**Figure 1:**
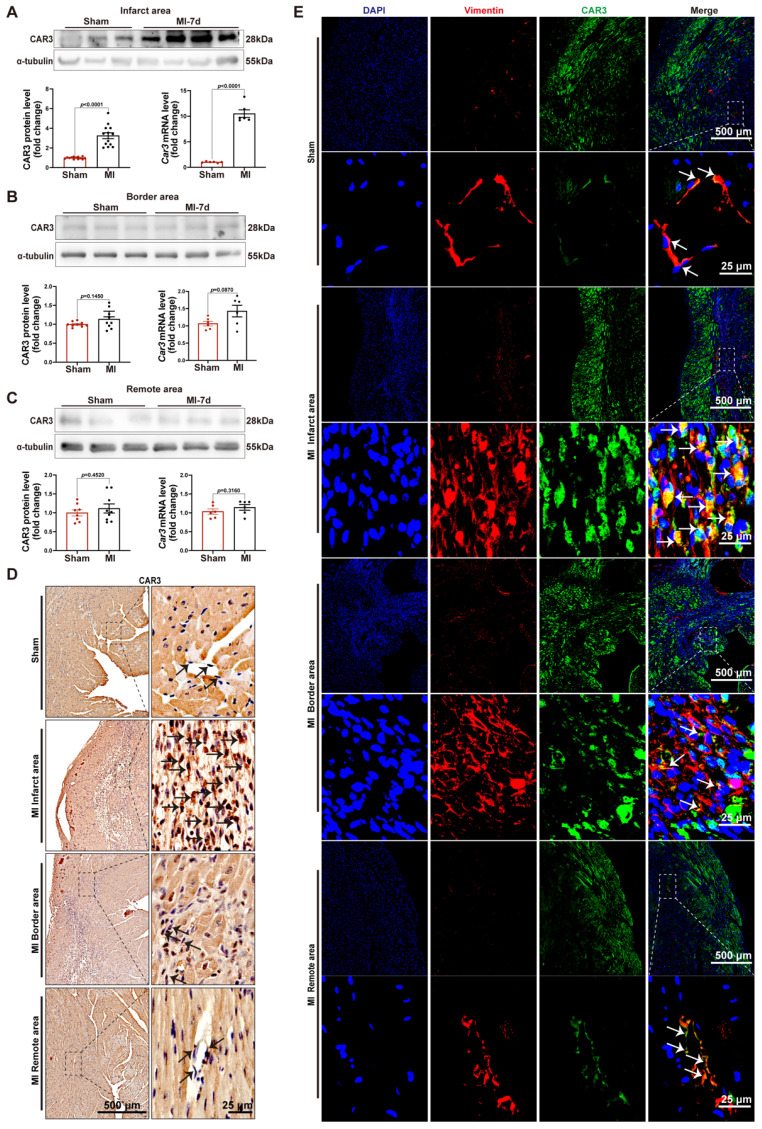
CAR3 expression is increased at the infarct area in response to myocardial infarction. A, Expression of CAR3 was measured using Western blot analysis (n = 12-13 mice per group) and RT q‐PCR (n = 6 mice per group) in the infarct area of WT mice at day 7 post-MI as well as in sham control. Results were normalized against α-tubulin and converted to fold induction relative to sham‐operated mice. B, CAR3 level was determined by Western blot analysis (n = 9 mice per group) and RT q‐PCR (n = 6 mice per group) in the border area of MI-operated hearts and their sham controls. Corresponding statistic of CAR3 was shown. C, CAR3 level was determined by Western blot analysis (n = 8-9 mice per group) and RT q‐PCR (n = 6 mice per group) in the remote area of MI-operated hearts and their sham controls. Corresponding statistic of CAR3 was shown. D, Immunohistochemistry for cardiac fibroblast CAR3 was indicated by black arrows in the sham or MI-operated heart tissue (n = 4 samples per group, the left scale bar =500 μm, the right scale bar =25 μm). E, Immunofluorescence co-staining for vimentin with CAR3 and DAPI in cardiac fibroblasts was shown by white arrows in the heart of WT mice at day 7 post-MI (n = 4 samples per group, the upper scale bar =500 μm, the lower scale bar =25 μm). CAR3 was labeled in green. vimentin was labeled in red. Nuclei stained with DAPI were blue. The data are expressed as mean ± SEM. The data shown in A-C were analyzed by Student's t-test. CAR3, Carbonic anhydrase 3; RT q‐PCR, real‐time quantitative polymerase chain reaction; WT, wild type; MI, myocardial infarction; DAPI, 4'6-diamidino-2-phenylindole; MI-7d, 7 days post myocardial infarction.

In order to investigate the role of CAR3 during myocardial infarction, we established animal model by ligation of LAD coronary artery with mice. As expected, the expression of CAR3 in myocardial tissue from sham mice was hardly observed, instead it could be detected easily using immunoblotting assay (Figure S1A). While the infarct region displayed increased expression post MI, with a peak at 7 days post MI and rapidly back to base level at day 14 post-MI (Figure 1A; Figure S1B, C). The expression pattern of *Car3* mRNA was similar to protein (Figure 1A; Figure S1C). We subsequently detected the expression of Carbonic anhydrase 2 (CAR2), which shares similar sequence identity and structure with CAR3 but is well known for its efficient and specific activity of CO_2_ hydration-dehydration reaction. The data found that CAR2 expression was considerably up-regulated at day 1 and persisted up to day 3 post myocardial infarction (Figure S1B, C), different from expression trend of CAR3. However, we observed no obvious expression change of CAR3 in the border and remote areas (Figure 1B, C; Figure S1D, E). Also, immunohistochemistry and immunofluorescence staining showed clearly higher CAR3 protein expression in the infarct myocardium than in sham controls, but no change for CAR3 levels in the border and remote regions of hearts post-MI (Figure 1D, E). Our data indicate a dramatical elevation of CAR3 in the infarct area of heart after MI, foretelling a potential role of CAR3 for this disease.

### Upregulation of CAR3 in CFs upon MI operation and TGF-β1 treatment *in vitro*

**Figure 2:**
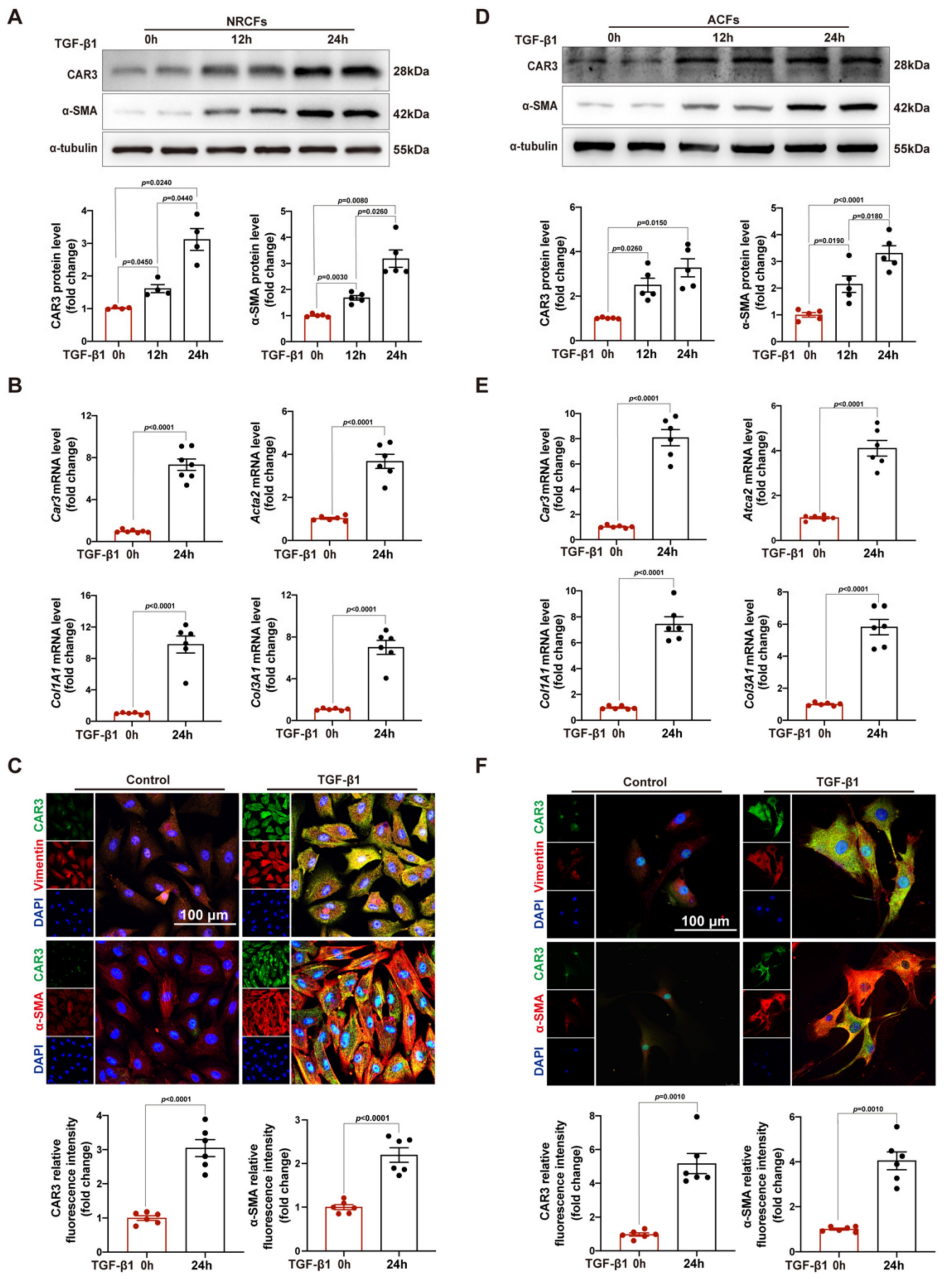
CAR3 level is up-regulated in TGF-β1-treated cardiac fibroblasts. A, Expression of CAR3 was determined using Western blot (n = 4-5 independent experiments per group) in TGF-β1‐treated NRCFs. Results were normalized against α-tubulin and converted to fold induction relative to control‐treated group. B, NRCFs were administrated by TGF-β1 or not for 24h. Relative mRNA expressions of *Car3*, *Acta2*, *Col1A1*and *Col3A1* were examined by RT q‐PCR (n = 6 independent experiments per group). Corresponding statistics were shown. C, Representative CAR3 immunofluorescence images of NRCFs treated with TGF-β1 or Control. CAR3 was labeled in green. Fibroblast markers α-SMA and vimentin were labeled in red. Nuclei stained with DAPI were blue. Fluorescence intensity of CAR3 and α-SMA staining was determined and expressed as fold change relative to control group (n = 6 independent experiments per group, scale bar = 100 μm). D, CAR3 and α-SMA Western blot bands (n = 5 per group) were shown in ACFs after stimulation with TGF-β1 or control. Corresponding statistics of CAR3 and α-SMA were shown. E, Relative mRNA expressions of *Car3*,* Acta2*, *Col1A1*and *Col3A1* were examined by RT q‐PCR (n = 6 per group) in ACFs treated by TGF-β1 for 24h. F, IF co-staining for α-SMA or vimentin with CAR3 and DAPI in TGF-β1-treated ACFs. Fluorescence intensity was measured and the results were presented as fold change against the corresponding controls (n = 6 independent experiments per group, scale bar = 100 μm). Data are expressed as mean ± SEM. The data shown in A and B were analyzed by one-way ANOVA followed by Bonferroni post hoc test. Data shown in B, C, E, and F were analyzed by Student's t-test. TGF-β1, transforming growth factor-β1; NRCFs, neonatal rat cardiac fibroblasts; α-SMA, α-smooth muscle actin; *Acta2*, actin alpha 2, smooth muscle, aorta; *Col1A1*, collagen type I alpha 1 chain; *Col3A1*, collagen type III alpha 1 chain; ACFs, adult mouse cardiac fibroblasts.

After increase of CAR3 in infarcted heart was identified, we further identify the cellular specificity of CAR3 expression change in cardiac tissue after MI. IF staining showed that the CAR3 in infarct area was colocalized with vimentin, a specific marker of cardiac fibroblasts, while there was only sporadic colocalization in the border and remote areas post-MI or sham-operated controls (Figure 1E). We then detected the expression of CAR3 in cultured NRCMs, NRCFs isolated from neonatal rat hearts and HUVEC cell line, and Western blot analysis demonstrated that NRCMs and NRCFs expressed CAR3 moderately, while no CAR3 was observed in cultured HUVECs (Figure S2A). To simulate ischemia condition, we cultured the cells by deprivation of glucose and oxygen, and observed no significant alteration of CAR3 and cTNT expression in NRCMs by either Western bolt analysis, RT-qPCR study, or IF staining (Figure S2B-D). We also observed no distinct expression difference for CAR3 and α-SMA in NRCFs and ACFs regardless exposed to hypoxia treatment or not (Figure S2E, F). While the expression of CAR3 markedly increased in NRCFs treated with TGF-β1, accompanied by increased expression of α-SMA (Figure 2A, B), and collagen-related genes such as *Col1A1* and *Col3A1* as well (Figure 2B). IF analysis further confirmed the colocalization of CAR3 with vimentin or α-SMA in NRCFs (Figure 2C). In addition, ACFs from WT mice displayed similar results by Western blotting analysis, RT-qPCR study and immunofluorescence staining (Figure 2D-F). While CAR2 protein levels in NRCFs and ACFs showed no dramatic changes between TGF-β1-treated groups and their controls (Figure S2G, H). These results confirmed specific CAR3 upregulation in cardiac fibroblasts but not cardiomyocytes in infarct area post MI.

### CAR3 deficiency impaired cardiac repair and aggravated cardiac dysfunction 7d post-MI

**Figure 3:**
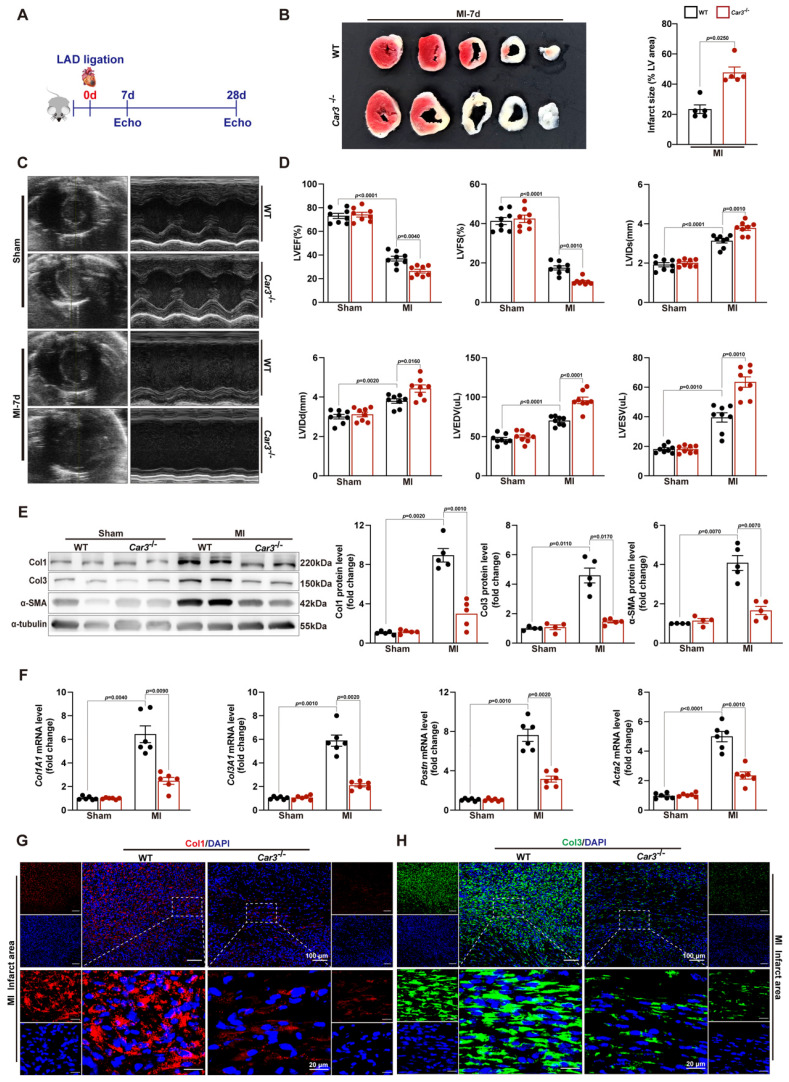
CAR3 deficiency impaired cardiac wounding healing and exacerbated cardiac dysfunction 7d after MI. A, Schematic protocol of the experimental approach. B, The infarct size of WT and *Car3*‐deficient mouse heart at 7 days after MI operation was determined by TTC staining and expressed as the percentage of infarct over ventricular area (n = 5 mice per group). C, Representative serial echocardiography via short axis acquired at 7d post-MI and relative quantifications (n = 8 mice per group). D, Cardiac function parameters, namely LVEF, LVFS, LVIDs, LVIDd, LVEDV, and LVESV were measured by echocardiography in indicated groups (n = 8 mice per group). E, Representative Western blot analysis and quantitative results of Col1, Col3, and α-SMA. Results were normalized against α-tubulin and converted to fold induction relative to their respective controls (n = 4-5 mice per group). F, Transcription levels of *Col1A1*,* Col3A1*, *postn*, and *Acta2* were measured by RT q‐PCR in the cardiac infarcted tissues of the indicated groups (n = 6 mice per group). G, Representative micrographs of Col1 expression by immunofluorescence staining in the infarct size at day 7 post-MI (n = 4 mice per group, the upper scale bar =100 μm, the lower scale bar =20 μm). H, Representative images of Col3 expression by IF in the infarct area 7 days post-MI (n = 4 mice per group, the upper scale bar =100 μm, the lower scale bar =20 μm). Data are presented as mean ± SEM, by one-way ANOVA followed by Bonferroni post hoc test. TTC, Triphenyltetrazolium chloride staining; LVEF, left ventricular ejection fraction; LVFS, left ventricular fractional shortening; LVIDs, left ventricular end‐systolic diameter; LVIDd, left ventricular end‐diastolic diameter; LVEDV, left ventricular end-diastolic volume; LVESV, left ventricular end-systolic volume.

The altered expression of CAR3 in myocardial tissue and TGF-β1-treated fibroblasts suggested its underlying role in the regulation of cardiac repair post-MI. Then, we subjected *Car3*‐deficient mice and their WT littermates to MI model. Echocardiographic parameters indicated a similar cardiac phenotype of *Car3*‐deficient mice and WT littermates at baseline (Table S2). However, triphenyltetrazolium chloride (TTC) staining revealed that CAR3 deficiency considerably enlarged left ventricular infarct area 7 days after MI surgery (Figure 3B). Similarly, cardiac function assessed by ultrasound showed a significantly worsen deterioration of left ventricular ejection fraction (LVEF) and fractional shortening (LVFS) in *Car3*‐deficient mice subjected to LAD ligation, and other parameters such as LV internal diameter at end diastole (LVIDd) and at end systole (LVIDs), LV end-diastolic volume (LVEDV) and end-systolic volume (LVESV), also suggested aggravated LV dilatation (Figure 3C, D).

To explore the effects of CAR3 on cardiac healing post-MI, the dynamic change and composition of the ECM in infarcted myocardium were investigated. We found that on day 7 post MI, α-SMA, Col1 and Col3 were upregulated in infarct zone, whereas CAR3 deficiency largely obviated the up-regulated expression (Figure 3E). In addition, CAR3 deficiency dampened the mRNA levels of ECM-related genes *Col1A1*, *Col3A1*, *Postn* and *Acta2* after myocardial infarction (Figure 3F). Consistently, IF staining also presented remarkable accumulation of Col1 and Col3 in the infarcted hearts of WT mice, but dramatic restrain of Col1 and Col3 increase in *Car3*-deficient mice (Figure 3G, H). Collectively, these results reveal that CAR3 deficiency worsened MI-induced cardiac dysfunction and infarct size, possibly by impairing deposition of collagen proteins, supporting a beneficial role for CAR3 in the proliferative phase of infarct healing.

### CAR3 deficiency contributed infarct size enlargement and heart failure

**Figure 4:**
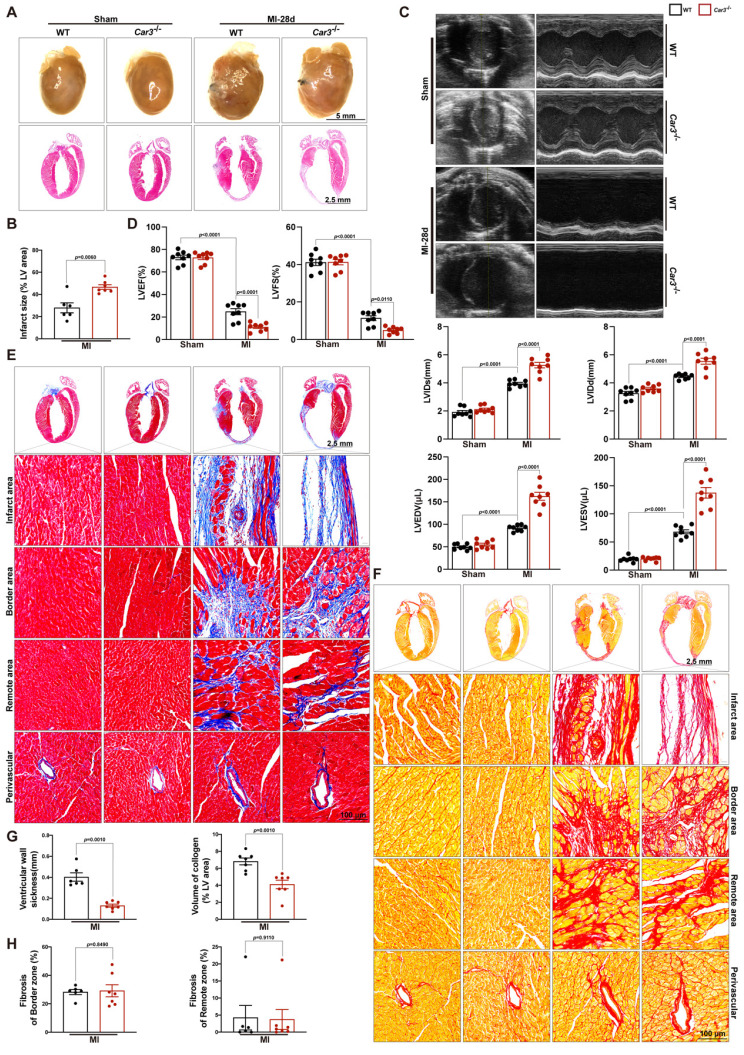
CAR3 deficiency enlarged infarct size and aggravated heart failure 28d post-MI. A, Representative pictures of the whole heart and HE staining of WT and *Car3*‐deficient mice after Sham or MI operation for 4 weeks (n = 6-7 mice per group). B, Infarct size was measured as the percentage of infarct over ventricular areas. C, Representative echocardiograms via short axis acquired from WT and *Car3*-knockout mice at 28 days after MI or sham operation and relative quantifications (n = 8 mice per group). D, Echocardiographic parameters of cardiac function, namely LVEF, LVFS, LVIDs, LVIDd, LVEDV, and LVESV, were measured in the indicated groups 4 weeks after MI (n = 8 mice per group). E, Representative photographs of Masson's trichrome staining in sections of hearts obtained from WT and *Car3*-deficient mice at day 28 after LAD ligation (n = 6-7 mice per group, the upper scale bar =2.5 mm, the lower scale bar =100 μm). F, Picrosirius red staining was performed to examine collagen density in the scar of different groups 4 weeks post-MI (n = 6-7 mice per group). G, Quantitative assessment of the ventricular wall thickness as well as the volume of collagen in the infarct area at 28d after MI (n=6-7 mice per group). H, Quantification of fibrotic areas in the border zone and remote zone at 28d post-MI in indicated groups (n=6-7 mice per group). The data are presented as mean ± SEM. B and D were analyzed by one-way ANOVA followed by Bonferroni post hoc test; G and H were analyzed by Student's *t* test.

Perturbed deposition of new structural matrix because of inadequate wound healing post-MI may lead to lethal cardiac rupture, enlarged infarct area and severe late-onset heart failure^7^. To test the effects of CAR3 deficiency on long-term cardiac function, we monitored cardiac function and pathological change 28 days after LAD ligation. Heart images and HE staining demonstrated that left ventricular scar formation in *Car3*-deficient mice was dramatically increased compared with WT mice (Figure 4A, B).

Echocardiographic parameters showed that permanent ischemia injury induced more deteriorating heart failure in *Car3*-knockout mice, testified by notably diminished LVEF and LVES and remarkably expanded LVIDs, LVIDd, LVESV and LVEDV (Figure 4C, D). Moreover, Masson trichrome and picrosirius red staining revealed that CAR3 deficiency strikingly inhibited the accumulation of collagen in infarct area 28 days after myocardial infarction, accompanied by distinctly thinner ventricular wall compared with WT mice (Figure 4E-H). Impressively, no significant difference of cardiac fibrosis in border zone, interstitial fibrosis in the remote zone or perivascular fibrosis was detected between two groups (Figure 4E-H). Thus, our studies indicate that CAR3 deficiency aggravated cardiac infarct size expansion and heart failure post-myocardial infarction.

### CAR3 deficiency restrained wound healing and cardiac fibroblasts activation

**Figure 5:**
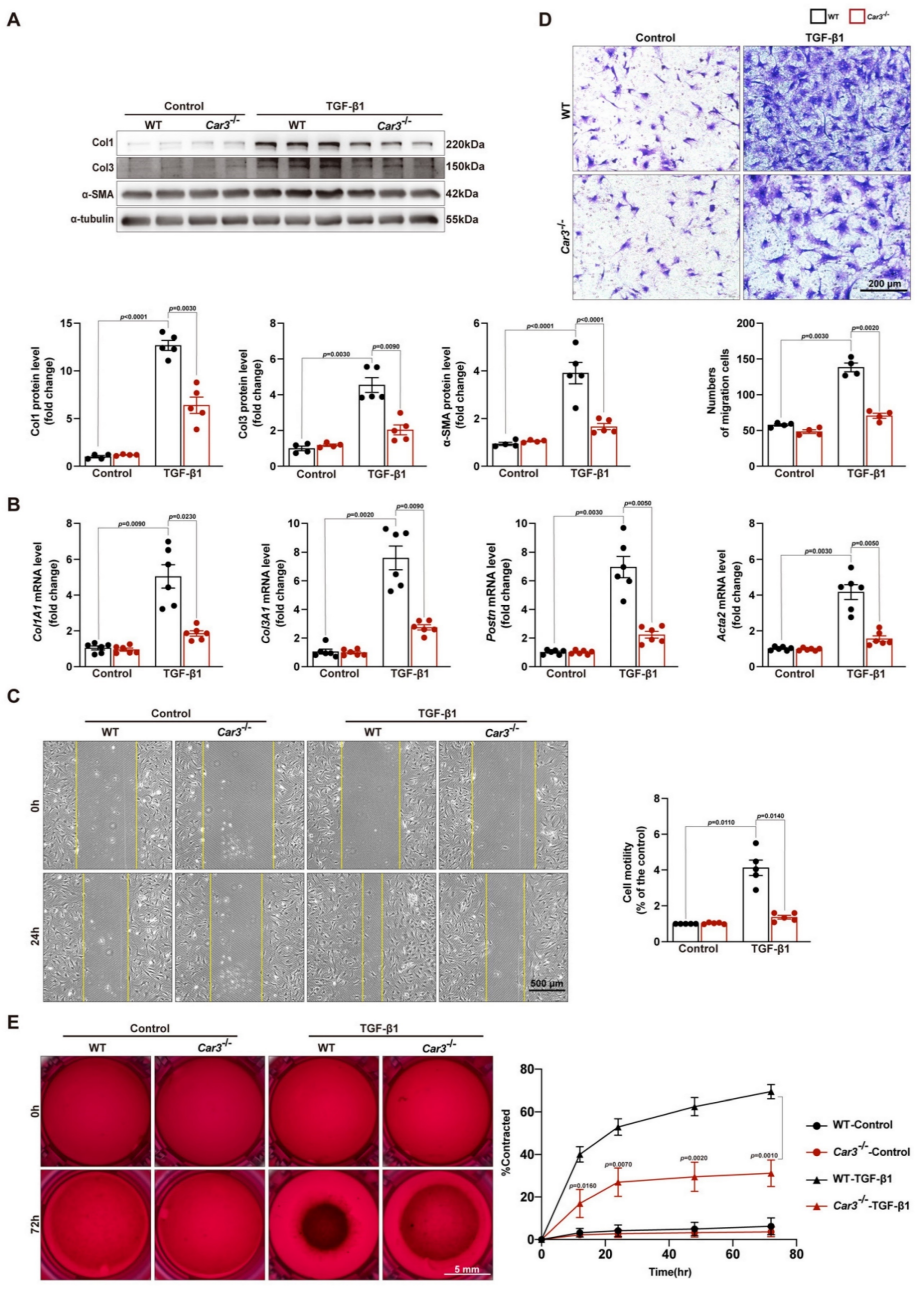
CAR3 deficiency weakened wound healing and cardiac fibroblasts activation *in vitro*. A, Western blot analysis and quantitative results of Col1, Col3, and α-SMA in ACFs treated with TGF-β1 for 24h isolated from WT and *Car3*-dificient mice. Results were normalized against α-tubulin and converted to fold induction relative to their respective controls (n = 5 independent experiments per group). B, RT q‐PCR was used to determine the transcription levels of* Col1A1*, *Col3A1*, *postn*, and* Acta2* in the indicated groups (n = 6 independent experiments per group). C, The migratory capacity of fibroblasts from WT and *Car3*-dificient mice respectively was assessed by the covering area of the scratch after administration with TGF-β1 for 24 h (n = 4 independent experiments per group, scale bar = 500 μm). D, Representative images of transwell assay after stimulation with TGF-β1 for 24h (n = 5 independent experiments per group, scale bar = 200 μm). E, Representative experiments of collagen gel contraction assay were investigated at different time points (n = 6 independent experiments per group, scale bar = 5 mm). Data are presented as mean ± SEM. The data shown in A-D were analyzed by one-way ANOVA followed by Bonferroni post hoc test. E was analyzed by two-way ANOVA followed by Bonferroni post hoc test.

Cardiac fibroblasts are key cells for replacing damaged and lost cardiomyocytes and prominent for contributing to cardiac repair after the acute injury period post-MI^10^,^43^. The decreased ECM deposition in the infarct area of *Car3*-dificient mice indicated fibroblasts activation disorder. To further investigate the effects of CAR3 on fibroblast activation, ACFs isolated from mice were cultured and treated with TGF-β1 (10ng/ml). TGF-β1 stimulation could clearly activate fibroblasts from WT mice based on up-regulation of protein levels of α-SMA, Col1 and Col3 as well as mRNA levels of *Col1A1*, *Col3A1*, *Postn* and *Acta2* (Figure 5A, B). In contrast, CAR3 deficiency obviously obviated the increase of α-SMA and collagen proteins (Figure 5A, B). Similarly, enhanced migratory capacity of fibroblasts from WT mice upon TGF-β1 stimulation were abolished by CAR3 deficiency based on transwell and wound-healing assays (Figure 5C, D). Gel contraction in fibroblasts lacking CAR3 was also dramatically hindered compared to activated myofibroblasts from WT mice (Figure 5E). On the whole, these results suggest that CAR3 deficiency significantly blunted TGF-β1 induced transformation of quiescent cardiac fibroblasts to myofibroblast based on collagen synthesis, cell migration and gel contraction.

### CAR3 mediated cardiac fibroblasts activation by stabilizing inhibitory Smad7

**Figure 6:**
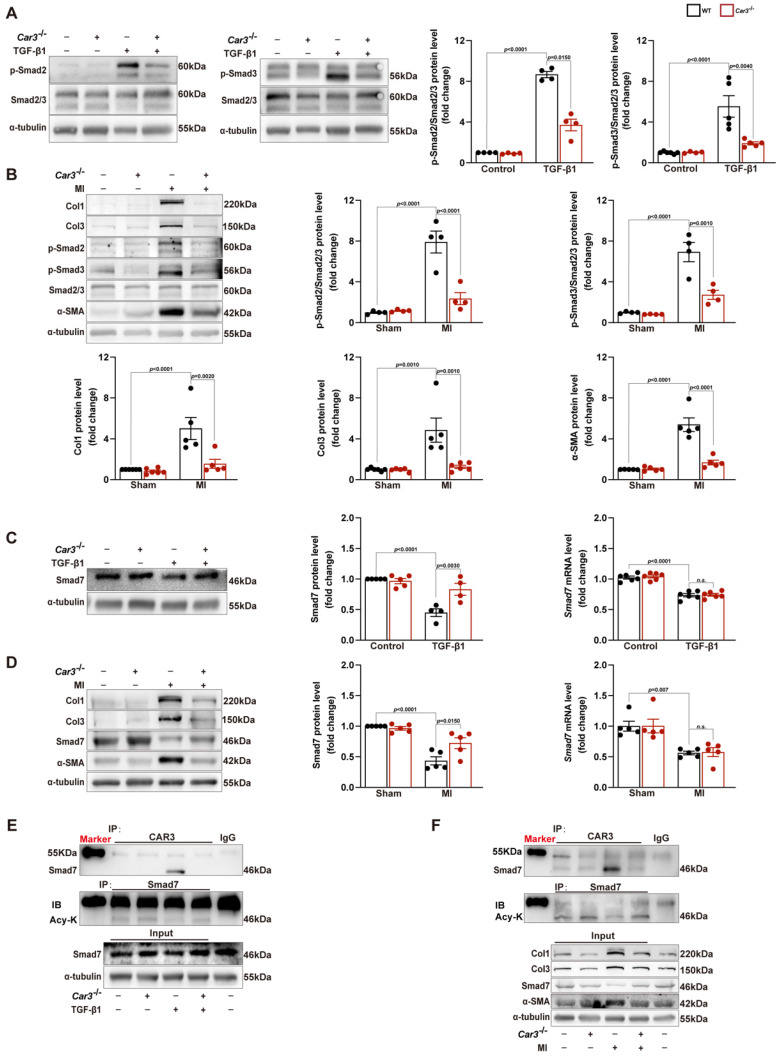
CAR3 deficiency inhibited the activation of TGF-β-Smad2/3 signaling pathway via mediating the stability of Smad7. A, Western blot analysis and statistical results of the levels of phosphorylated Smad2 and Smad3 in ACFs treated with TGF-β1 for 6h were examined and shown (n = 4 per group). B, Protein levels of phosphorylated Smad2 and Smad3 as well as Col1, Col3 and α-SMA were determined by Western blot (n = 4‐5 mice per group) in the infarcted heart of MI‐treated mice. C, Expression of Smad7 was measured using Western blot and RT q‐PCR (n = 4‐5 independent experiments per group) in cultured ACFs from WT and *Car3*-deficient mice 6h after TGF-β1 treatment. Results were normalized against α-tubulin and converted to fold induction relative to control‐treated group. D, Representative Western blots and statistical results of Smad7 in the infarct zone 7d post-MI (n = 5 mice per group). mRNA level of *Smad7* was detected by RT q‐PCR in the indicated groups (n = 5 mice per group). E, Interaction of CAR3 with Smad7 in cultured ACFs of different groups was determined by immunoprecipitation with anti-CAR3 antibody followed by immunoblot with anti-Smad7 antibody. Following immunoprecipitation of Smad7, the acetylation of Smad7 was detected with anti-acetylated lysine antibody (Acy-K). IgG as a negative control (n = 3). F, Co-immunoprecipitation and Western blots of interaction of CAR3 with Smad7 and the change of Smad7 acetylation in infarcted heart tissues. IgG as a negative control (n = 3). The data are shown as the means ± SEM. The data shown in A, B, C, and D were analyzed by one-way ANOVA followed by Bonferroni post hoc test. Acy-K, acetylated lysine.

Overwhelming evidence indicated that TGF-β and its downstream Smad-dependent signaling is a key inducer of fibroblast-to-myofibroblast conversion and ECM production^17^,^44^. To explore the molecular mechanism underlying CAR3-dependent fibroblast activation, we first investigated the expression of TGFβR1 and TGFβR2. Although TGFβR1 and TGFβR2 were increased in the infarct area of hearts after MI and in TGF-β1 stimulated CFs, CAR3 deficiency exerted no effects on their expressions (Figure S5A-D). We then explored the effects of CAR3 on TGF-β-Smad2/3 signaling. Up-regulation of phosphorylation of Smad2 and Smad3 were observed in ACFs treated with TGF-β1 as well as in infarcted cardiac tissues of WT mice 7d after MI, and CAR3 knockout strikingly inhibited the activation signal of Smad2 and Smad3 (Figure 6A, B). Smad6 and Smad7 (I-Smads) are well-studied negative signal regulators in TGF-β pathways, including phosphorylation of Smad2/3^45^,^46^. As Smad6 preferentially inhibits bone morphogenetic protein (BMP)-induced Smad signaling^47^ while Smad7 restrains both BMP- and TGF-β-induced Smad signaling^48^, we subsequently detected the role of CAR3 on Smad7 expression change. After exposed to TGF-β1 treatment for 6 hours, Smad7 protein level was notably decreased in CFs from WT mice, which could be reversed when lacking CAR3 (Figure 6C). In parallel, CAR3 deficiency also dramatically weakened the reduction of Smad7 protein expression in infarcted cardiac tissue (Figure 6D)*.* However, no significant expression change of Smad7 transcription level were observed both *in vitro* and *in vivo* (Figure 6C, D), which prompted us to explore how CAR3 affected Smad7 stability or turnover.

So we first evaluated the interaction between CAR3 and Smad7 in cardiac fibroblasts lysates by co-immunoprecipitation assays, and an increased association of CAR3 and Smad7 was observed in ACFs if treated with TGF-β1 (Figure 6E). As acetylation modification promotes Smad7 protein stability while TGF-β signaling inhibits its acetylation^49^,^50^, we then explored the function of CAR3 on Smad7 acetylation.

**Figure 7:**
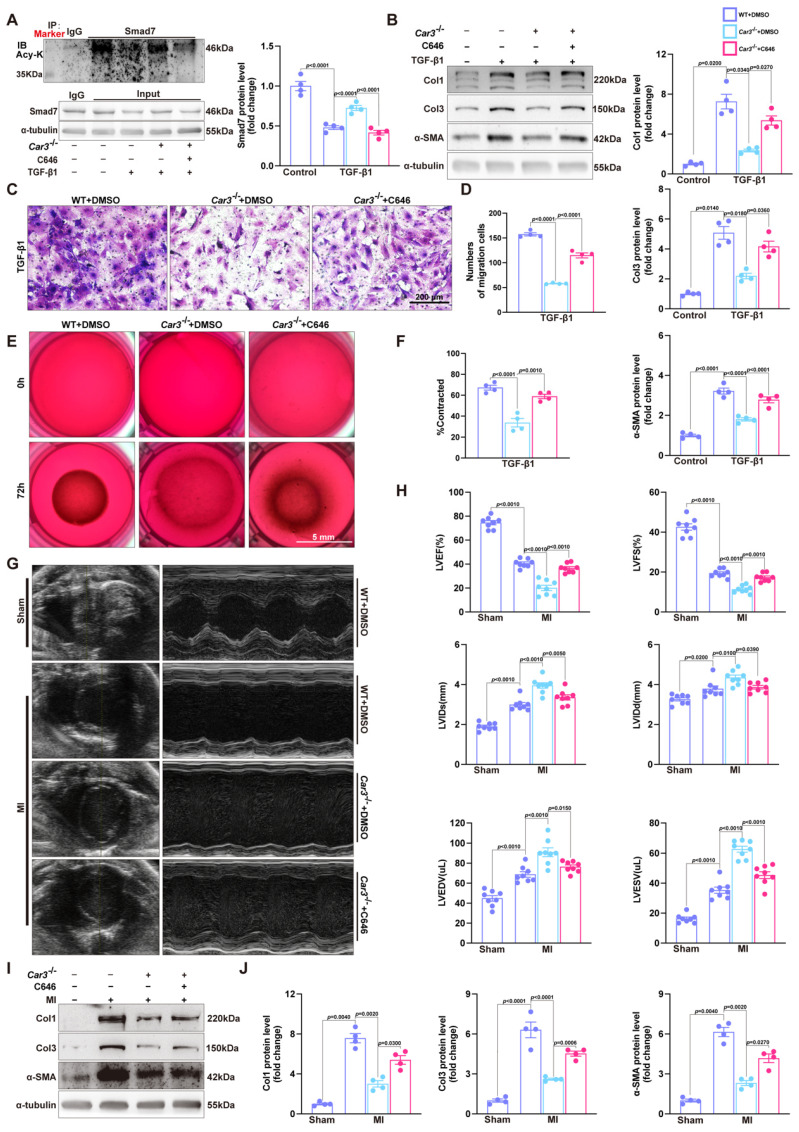
C646-pretreatment reversed CAR3 deficiency induced suppression of fibroblasts activation *in vitro* and impairment of cardiac healing and cardiac dysfunction *in vivo*. A, WT, *Car3*-knockout, or C646-preincubated *Car3*-knockout ACFs were stimulated with TGF-β1 for 6 h. Expressions of Smad7 and its acetylation were determined using Western blot (n = 4 independent experiments per group). B, ACFs were preincubated with DMSO or C646 followed by treatment with TGF-β1 for 24 h. Protein levels of Col1, Col3, and α-SMA were detected by Western blot (n = 4 per group). C, Representative images of transwell assay in indicated groups (n = 4 independent experiments per group, scale bar = 200 μm). D, Quantitative results for the migration capacity were measured. E, Representative experiments of collagen gel contraction assay in ACFs preincubated with DMSO or C646 followed by TGF-β1 treatment (n = 4 independent experiments per group, scale bar = 5 mm). F, Quantitative results for collagen gel contraction ability in indicated groups. G, WT, *Car3*-knockout, or C646-pretreated *Car3*-knockout mice were followed to induce mouse MI model. Representative serial echocardiography acquired by short axis at 7d post-MI and relative quantifications (n = 8 mice per group). H, Cardiac function parameters (LVEF, LVFS, LVIDs, LVIDd, LVEDV, and LVESV) were measured by echocardiography in indicated groups (n = 8 mice per group). I, Representative Western blot analysis (n = 4 mice per group) and quantitative results of Col1, Col3, and α-SMA. J, Statistical results of Col1, Col3, and α-SMA in the infarct zone 7d post-MI. Data are presented as mean ± SEM, by one-way ANOVA followed by Bonferroni post hoc test. DMSO, Dimethyl sulfoxide.

Co-immunoprecipitation and Western blot analysis demonstrated that TGF-β1 treatment caused decrease of Smad7 acetylation in CFs from WT mice, while CAR3 deficiency increased the acetylation of Smad7 (Figure 6E). Unsurprisingly, *in vivo* investigations showed that the enhanced association of CAR3 and Smad7 induced in MI was revoked by CAR3 deficiency, so do reduction of Smad7 acetylation (Figure 6F). To further assessed whether CAR3 mediated Smad7 protein stability through regulating acetylation, compound C646 experiments were performed to inhibit p300^42^, which is the acetyltransferase responsible for Smad7 acetylation. C646 treatment of fibroblast cells restrained the increased acetylation of Smad7, thus inhibiting the upregulation of Smad7 protein level induced by CAR3 deficiency (Figure 7A; S6A, B). And accordingly, inhibition of fibroblast activation was released in CAR3 deficiency condition (Figure 7B-F). This function was mirrored by alleviated CAR3 deficiency-exerted impaired cardiac healing, and improved cardiac function accompanied with increased collagen synthesis in the infarcted hearts 7d post-MI (Figure 7G-J). These data demonstrate that CAR3 plays a role in regulating Smad7 protein stability by modifying p300-dependent acetylation.

### CAR3 overexpression promoted wound healing and cardiac fibroblasts activation *in vitro*

**Figure 8:**
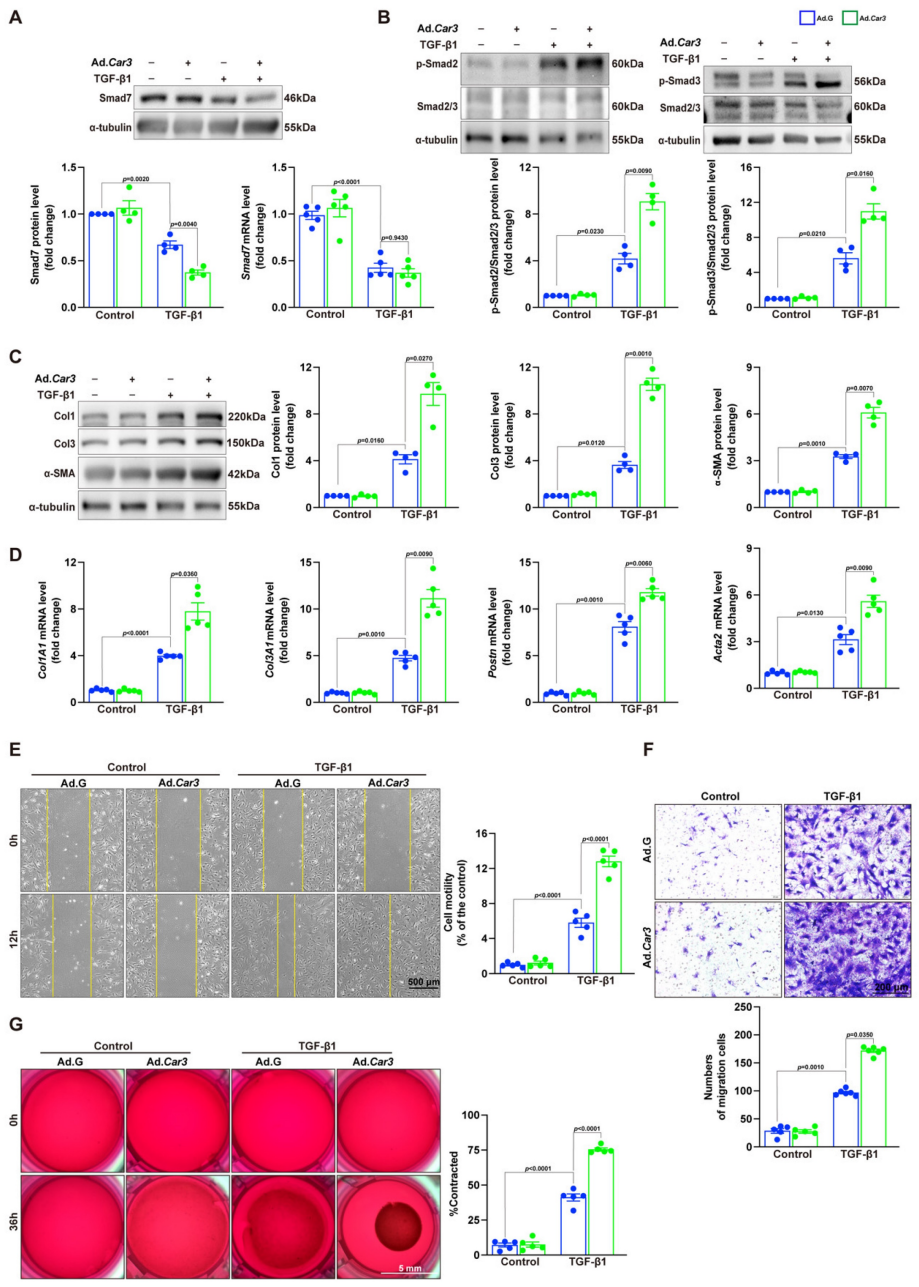
CAR3 overexpression promoted cardiac fibroblasts activation and wound healing *in vitro*. A, ACFs isolated from WT mice were infected with Adv-*Car3* (Ad.*Car3*) or Adv-GFP (Ad.G) for 48 h before the cells were treated with TGF-β1 for 6 h. Expression of Smad7 was determined using Western blot and RT q‐PCR (n = 4‐5 independent experiments per group). B, Expression level of phosphorylated Smad2 and Smad3 in ACFs were examined by Western blot. Corresponding statistics were shown (n = 4 per group). C, ACFs were infected with Adv-*Car3* or Adv-GFP for 48 h followed by treatment with TGF-β1 for 24 h. Protein levels of Col1, Col3, and α-SMA and statistical results were detected by Western blot (n = 4 per group). D, The transcription levels of *Col1A1*, *Col3A1*, *postn*, and *Acta2* were determined by RT q‐PCR in the indicated groups (n = 4 per group). E, Quantitative results for the migration ability of ACFs in indicated groups was measured by the covering area of the scratch (n = 5 independent experiments per group, scale bar = 500 μm). F, Representative images of transwell assay in ACFs transduced with Adv-*Car3* or Adv-GFP for 48 hours followed by TGF-β1 treatment (n = 5-6 independent experiments per group, scale bar = 200 μm). G, Collagen gel contraction assay was carried out to test the contraction capacity of ACFs after 36h administration with TGF-β1 in indicated groups (n = 6 independent experiments per group, scale bar = 5 mm). Data are presented as mean ± SEM, by one-way ANOVA followed by Bonferroni post hoc test. GFP, green fluorescent protein.

To further confirm the effects of CAR3 on fibroblast activation, we conducted experiments with Adv-GFP or Adv-*Car3* to overexpress CAR3 or not (Figure S7A-C). Western blotting revealed that CAR3 overexpress in fibroblasts exerted a further reduction of Smad7 protein level under TGF-β1 treatment, unsurprisingly, no significant difference of *Smad7* mRNA level was detected (Figure 8A). We then observed that fibroblasts infected with Adv-*Car3* signally increased the expression of phosphorylation of Smad2 and Smad3 compared to Adv-GFP group after TGF-β1 treatment for 6 hours (Figure 8B). Accordingly, the expression of α-SMA and related collagen proteins were promoted further in CAR3 overexpression group upon TGF-β1 stimulation (Figure 8C, D). Moreover, overexpression of CAR3 promoted both cellular migration and contraction of fibroblasts (Figure 8E-G). Overall, these data confirm that CAR3 plays a key role in activation of cardiac fibroblasts and synthesis of ECM.

## Discussion

In the present work, we report a cardioprotective role of CAR3 in cardiac repair post-MI. The up-regulation of CAR3, mainly in fibroblasts, is responsive upon myocardial injury, and is beneficial for cardiac healing, while CAR3 deficiency in mice significantly impaired cardiac wounding healing, leading to deteriorated cardiac function and increased infarct size. Mechanistically, CAR3 involved in conversion of fibroblasts to myofibroblasts via activation of TGF-β-Smad2/3 signaling pathway through regulating acetylation of Smad7. Thus our current study provides evidence that CAR3 promotes CF activation and potentially anticipates a promising therapeutic target for cardiac repair post-MI.

Substantial evidence has verified the effects of CAR3 in regulating not only physiological processes (fatty acid metabolism and oxidative stress response) but pathological conditions (muscle dysfunction, cancer progression, and autoimmune diseases)^19^,^22^,^51^-^53^. Dramatic elevation of CAR3 was observed in multiple cardiovascular biological processes, suggesting the potential involvement of CAR3 in the pathogenesis of cardiovascular diseases^33^-^36^,^54^. However, these studies failed to explore whether and how CAR3 works during myocardial ischemia injury. In this study, we find that CAR3 plays a critical role on cardiac fibroblasts during cardiac wound healing post-MI. CAR3 deficiency dramatically hampers transformation of cardiac fibroblast to myofibroblast, while CAR3 overexpression facilitates it.

As a key source of ECM, cardiac fibroblasts involve in controlling the homeostasis of microenvironment in mammalian heart and maintaining cardiac architecture and performance post-MI^14^,^55^. Upon exposure to MI injury, timely and sufficient transformation of fibroblasts to myofibroblasts is vital to promote cardiac repair, prevent cardiac rupture, and alleviate later myocardial remodeling and cardiac dysfunction^10^,^43^,^56^,^57^. During ischemic myocardial injury, TGF-β and its downstream signaling pathway is one of the most potent regulatory cascades for fibroblast differentiation^44^,^58^,^59^. TGF-β binds a heterodimeric receptor in the plasma membrane consisting of the TGF-β receptor type 1 (TGFβR1) and 2 (TGFβR2), which subsequently activates canonical Smad or non-canonical signaling cascades^16^.

Canonical TGF-β-Smad2/3 signaling is well-documented for dominating myofibroblast activation and matrix protein synthesis^60^-^64^. Our data show that CAR3 deficiency notably suppresses activation of Smad2/3 signaling pathway in the infarct size of cardiac tissue post-MI and in cardiac fibroblasts under TGF-β1 treatment.

A variety of positive and negative regulators involves in regulating TGF-β-Smad2/3 signaling^16^. Among them, Smad7 has aroused great interest as a pleiotropic endogenous inhibitor that represses intracellular signaling through interactions with activated type I receptors (TGFβRs) or R-Smads^46^. Our current study showed that Smad7 protein level was down-regulated in infarcted heart tissue post MI, while CAR3 deficiency reversed the decrease of Smad7 protein expression with no observable change of *Smad7* mRNA expression. We did observe an increased interaction of CAR3 and Smad7 both *in vivo* and *in vitro* suggesting a potential role of CAR3 on posttranslational modification of Smad7 protein. Modifications of Smad7 are associated with its stability and interaction with other proteins, which in turn regulates its functions^46^. Lys64 and Lys70 in the N terminus of Smad7 can be acetylated by acetyltransferase p300 and thus stabilizes Smad7, preventing it from subsequent ubiquitination degradation^46^,^49^. Our data showed that LAD-ligation and TGF-β1 treatment strikingly reduced Smad7 acetylation, while CAR3 deficiency restored the acetylation of Smad7. Meanwhile, C646, a specific inhibitor for p300 acetyltransferase, blunt CAR3 deficiency-exerted increase of Smad7 acetylation and freed restrain of fibroblast activation, and improved impaired cardiac repair and collagen synthesis. These results provide evidence supporting the hypothesis that CAR3 regulates the acetylation of Smad7 mediated by acetyltransferase p300. To our knowledge, this is the first study to identify CAR3 as a crucial regulator of cardiac fibroblasts activation via the TGF-β-Smad2/3 pathway and Smad7 protein as a target of CAR3.

However, some limitations exist in the current study, and need further research. The first one is what causes the increase of CAR3 expression in infarcted myocardial tissue. The second question is that we may need a cell-specific, like fibroblast-specific *Car3* deficient mice, to exclude the effects of CAR3 on cardiomyocytes during MI. The module of how CAR3 affects p300-mediated Smad7 acetylation is also not clear. Other interesting issue also include the individual role of CAR3 in other phases, which are necessary to further investigations about therapeutic time window for MI. Elucidating these issues may provide translational application for CAR3 in intervening cardiovascular disease.

## Supplementary Material

Supplementary materials and methods, figures and tables.

## Figures and Tables

**Figure 9:**
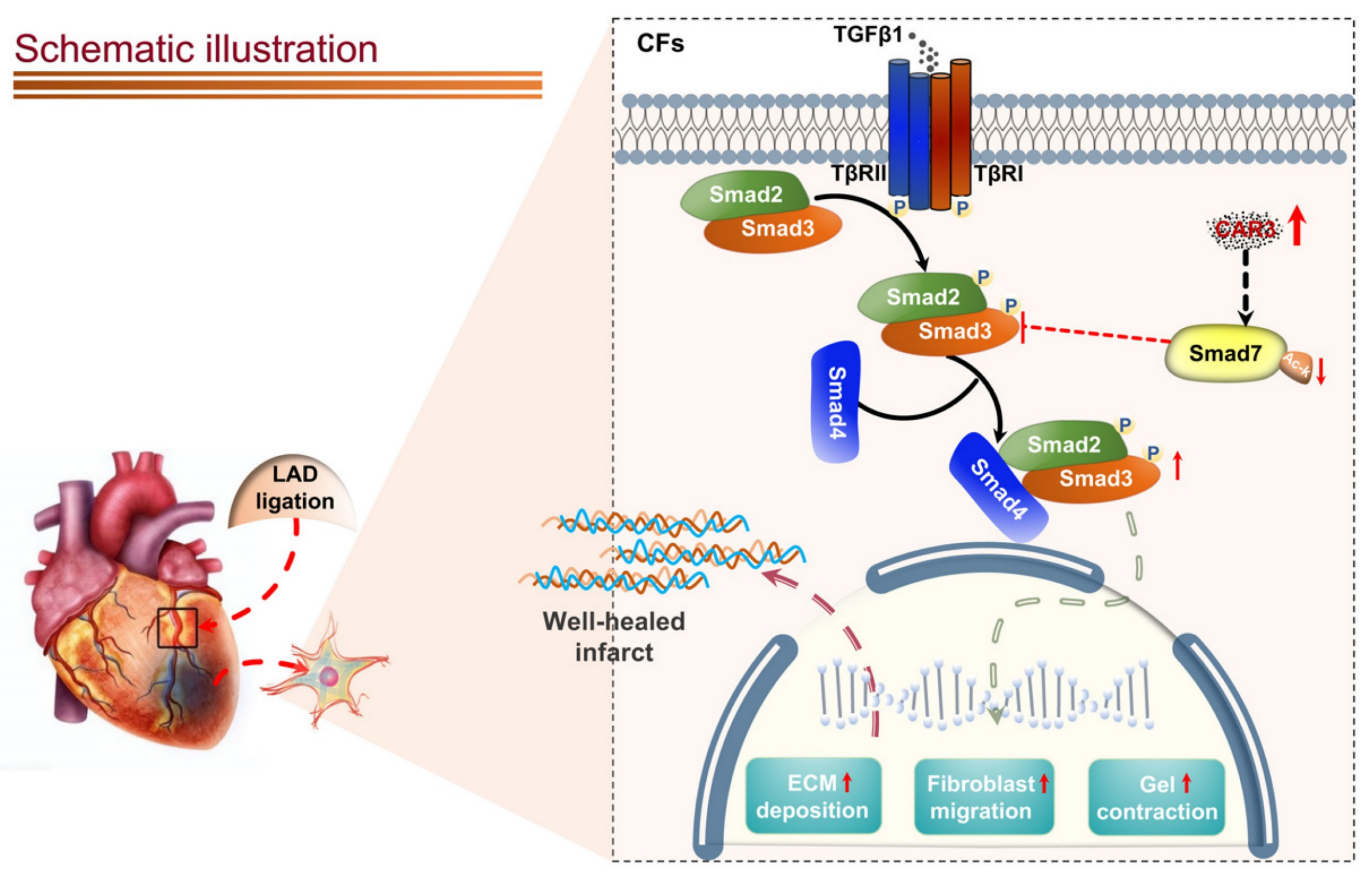
Schematic diagram depicting the role of CAR3 in cardiac repair post MI. CAR3 deficiency aggravated infarct size enlargement and cardiac dysfunction after myocardial infarction through inhibition of cardiac fibroblasts transformation. Mechanistically, CAR3 promotes fibroblast transformation by TGF-β-Smad2/3 signaling via repressing acetylation of inhibitory Smad7, which ultimately improves timely cardiac repair post MI.

## Abbreviations

MI: myocardial infarction
CF: cardiac fibroblast
CAR3: carbonic anhydrase 3
ACF: adult mouse primary cardiac fibroblast
CHD: Coronary heart disease
ECM: extracellular matrix
⍺-SMA: α-smooth muscle actin
DCM: dilated cardiomyopathy
HCM: hypertrophic cardiomyopathy
WT: wild‐type
LAD: left anterior descending artery
LVID: left ventricular internal diameter
LVEF: left ventricular ejection fraction
LVFS: left ventricular fractional shortening
LVEDV: left ventricular end-diastolic volume
LVESV: left ventricular end-systolic volume
TTC: triphenyltetrazolium chloride
NRCF: neonatal rat cardiac fibroblasts
NRCM: neonatal rat cardiac myocyte
PBS: phosphate buffered saline
FBS: fetal bovine serum
HUVEC: human umbilical vein endothelial cells
Adv: recombinant adenovirus
PVDF: polyvinylidene difluoride membranes
ECL: enhanced-chemiluminescent system
RT q‐PCR: real‐time quantitative polymerase chain reaction
IF: immunofluorescence
DAPI: 4′,6-diamidino-2-phenylindol
HE: hematoxylin and eosin
ANOVA: analysis of variance
BMP: bone morphogenetic protein
TGFβR: TGF-β receptor
