# Supplementary material for: Carbonic Anhydrase 3 is required for cardiac repair post myocardial infarction via Smad7-Smad2/3 signaling pathway
Source: Int J Biol Sci. 2024 Feb 25;20(5):1796–814. doi: 10.7150/ijbs.91396 (PMC10929199; doi:10.7150/ijbs.91396)

SUPPLEMENTAL MATERIAL

Carbonic Anhydrase 3 is required for cardiac repair post myocardial infarction via Smad7-Smad2/3 signaling pathway

Yuanyuan Su[1#], Dongmei Shi[1#], Guofang Xia[1], Yujia Liu[2], Longwei Xu[3], Ling Dao[3], Xia Lu[1], Chengxing Shen[1✉], and Congfeng Xu[1,4✉]

1. Department of Cardiology, Shanghai Sixth People's Hospital Affiliated to Shanghai Jiao Tong University School of Medicine, Shanghai 200233, China.
2. Department of Neurology, Tongren Hospital Affiliated to Shanghai Jiao Tong University School of Medicine, Shanghai 200042, China.
3. Department of Cardiology, the First Affiliated Hospital of Zhengzhou University, Zhengzhou 450000, China.
4. Shanghai Key Laboratory of Sleep Disordered Breathing, Shanghai 200233, China.

Running Title: Carbonic Anhydrase 3 deficiency impairs cardiac repair.

[#]These authors contributed equally to this work.

Corresponding authors: Congfeng Xu: Email: cxu@shsmu.edu.cn, Tel/Fax: +86-21-24058721; Chengxing Shen: Email: shencx@sjtu.edu.cn, Tel/Fax: +86-21-24058441.

Supplementary Figures and Tables

Figure S1. The expression levels of CAR3 in normal and infarcted hearts post-MI. A, Protein expression of CAR3 in adult mice tissue was shown. B, The temporal protein expression patterns of CAR3 and CAR2 were detected in the infarct area by Western blot analysis (n = 4-6 mice per group) and RT q-PCR (n = 4-5 mice per group). C, Corresponding statistics of CAR3 and CAR2 were shown. D, CAR3 level at different time points was determined by Western blot (n = 4 mice per group) and RT q-PCR (n = 6 mice per group) in the border area of MI-operated hearts. E, Expression of CAR3 at different time points was measured using Western blot (n = 4-5 mice per group) and RT q-PCR (n = 4-5 mice per group) in the remote area of MI-treated hearts. The data are shown as the means ± SEM and analyzed by Student's t-test. CAR3, carbonic anhydrase 3; MI, myocardial infarction; CAR2, carbonic anhydrase 2; RT q-PCR, real-time quantitative polymerase chain reaction.

Figure S2. The expression of CAR3 showed no significant change in NRCMs exposed to hypoxia. A, Western blot bands of CAR3 in cultured NRCMs, NRCFs, and HUVECs. B, NRCMs were treated by hypoxia for 3h or 6h. CAR3 expression was examined by Western blot analysis (n = 5-6 per group) and RT q-PCR (n = 6 per group). C, Corresponding statistic of CAR3 was shown. D, IF co-staining for cTnT with CAR3 and DAPI in NRCMs exposed to hypoxia for 6h (n = 5 per group, scale bar = 20 μm). E, CAR3 level was determined by Western blot analysis in NRCFs treated with hypoxia for 12h or 24h (n = 4 per group). F, Expression of CAR3 was measured using Western blot analysis (n = 4 per group) in ACFs exposed to hypoxia for 12h or 24h. G. Protein level of CAR2 was detected by Western blot in cultured NRCFs treated with TGF-β1 (n = 6 per group). H, Expression of CAR2 was measured using Western blot in cultured ACFs treated with TGF-β1 (n = 6 per group). The data are shown as the means ± SEM. The data shown in C and E-H were analyzed by one-way ANOVA followed by Bonferroni post hoc test, and D was analyzed by Student's t-test. NRCMs, neonatal rat cardiomyocytes; NRCFs, neonatal rat cardiac fibroblasts; HUVECs, human umbilical vein endothelial cells; cTnT, cardiac troponins T; DAPI, 4'6-diamidino-2-phenylindole; TGF-β1, transforming growth factor-β1; ACFs, adult mouse cardiac fibroblasts.

Figure S3. Validation of *Car3*-dificient in mice. A, Schematic diagram of *Car3* gene-targeting strategy. B, Expression of CAR3 was measured using Western blot in normal (n = 8 mice per group) and infarcted hearts 7d post-MI from WT and *Car3*-deficient mice. C, Quantitative results for CAR3 in Western blot and RT q-PCR (n = 6 mice per group) were measured. Data are presented as mean ± SEM, and analyzed by Student's t-test.

Figure S4. The expression of CAR3 in cultured ACFs isolated from WT or *Car3*-knockout mice. A, Expression of CAR3 was measured by Western blot in ACFs from WT and *Car3*-deficient mice (n = 8 per group). B, Corresponding statistic of CAR3 was shown. Data are presented as mean ± SEM, and analyzed by Student's t-test. ACFs, adult mouse cardiac fibroblasts.

Figure S5. CAR3 deficiency exerted no significant effects on the expression of TGFβR1 or TGFβR2. A, Expressions of TGFβR1 was determined using Western blot (n = 4 per group) and RT q-PCR (n = 4 per group) in the infarct area of cardiac tissue post-MI. B, Expressions of TGFβR2 was measured by Western blot (n = 4 per group) and RT q-PCR (n = 4 per group) in the infarcted hearts after MI. Results were normalized against α-tubulin and converted to fold induction relative to control-treated group. C, TGFβR1 level was measured by Western blot (n = 4 per group) and RT q-PCR (n = 4 per group) in TGF-β1-treated ACFs. D, TGFβR2 level was determined using Western blot (n = 4 per group) and RT q-PCR

(n = 4 per group) in indicated groups. Data are presented as mean ± SEM, and analyzed by one-way ANOVA followed by Bonferroni post hoc test. TGFβR1, TGF-β receptor type 1; TGFβR2, TGF-β receptor type 2.

Figure S6. The expression of p300 in cultured ACFs and hearts from WT mice treated with DMSO or C646. A, Expressions of p300 was determined using Western blot (n = 4 independent experiments per group). B, Corresponding statistic of p300 was shown. C, Protein level of p300 in hearts from WT mice pretreated with DMSO or C646. D, Corresponding statistic of p300 was shown. Data are presented as mean ± SEM, and analyzed by Student's t-test. DMSO, Dimethyl sulfoxide.

Figure S7. The expression of CAR3 in cultured ACFs transduced with Adv-GFP or Adv-*Car3* to overexpress CAR3 for 48 hours. A, Protein level of CAR3 was measured by Western blot (n = 6 independent experiments per group). B, Transcription level of *Car3* was determined using RT q-PCR (n = 6 independent experiments per group). Data are presented as mean ± SEM, and analyzed by Student's t-test. GFP, green fluorescent protein.

Table S1. Primer sequences.

Gene	Forward primer (5'-3')	Reverse primer (5'-3')
Mus Car3	GCTTCACCTGGTTCACTGGAATCC	ACTCGCCTTTCTCCCGTCCTATC
Mus Car2	TCCCACCACTGGGGATACAG	CTCTTGGACGCAGCTTTATCATA
Mus Acta2	GTCCCAGACATCAGGGAGTAA	TCGGATACTTCAGCGTCAGGA
Mus Col1A1	GCTCCTCTTAGGGGCCACT	CCACGTCTCACCATTGGGG
Mus Col3A1	CTGTAACATGGAAACTGGGGAAA	CCATAGCTGAACTGAAAACCACC
Mus postn	CCTGCCCTTATATGCTCTGCT	AAACATGGTCAATAGGCATCACT
Mus β-actin	TATGCTCTCCCTCACGCCATCC	GTCACGCACGATTTCCCTCTCAG
Rat Car3	ACCGACTTCGCCAATTCCATCTTC	CTCAGCAGCATACTTCACTCCATCC
Rat Car2	TGTCAGCAGTGAGCAGATGTCTC	ACGCCAGTTGTCCACCATCAG
Rat Acta2	GCGTGGCTATTCCTTCGTGACTAC	CATCAGGCAGTTCGTAGCTCTTCTC
Rat Col1A1	TGTTGGTCCTGCTGGCAAGAATG	GTCACCTTGTTCGCCTGTCTCAC
Rat Col3A1	AGTCGGAGGAATGGGTGGCTATC	CAGGAGATCCAGGATGTCCAGAGG
Rat β-actin	GCTGTGCTATGTTGCCCTAGACTTC	GGAACCGCTCATTGCCGATAGTG

Table S2. Parameters in *Car3*-deficient (*Car3*$^{-/-}$) mice and their wild-type (WT) littermates at 7 days after MI operation.

/	Sham		MI	
Parameter	WT (n=8)	*Car3*$^{-/-}$ (n=8)	WT (n=8)	*Car3*$^{-/-}$ (n=8)
HR (beats/min)	475.83±12.94	484.01±14.05	481.70±19.64	481.12±15.72
LVIDd (mm)	3.00±0.30	3.11±.32	3.79±0.30	4.43±0.49
LVIDs (mm)	1.92±0.28	2.00±0.17	3.13±0.29	3.78±0.31
LVEDV (ml)	46.60±5.86	49.58±6.40	69.86±5.20	95.82±10.88
LVESV (ml)	18.15±2.50	18.01±2.36	39.49±8.30	63.51±9.33
LVEF (%)	73.07±5.72	74.10±5.36	36.96±5.41	26.38±4.18
LVFS (%)	41.28±4.84	42.43±4.94	17.49±2.86	10.54±1.51

HR, heart rate; LVIDd, left ventricular end-diastolic diameter; LVIDs, left ventricular end-systolic diameter; LVEF, left ventricular ejection fraction; LVFS, left ventricular fractional shortening; LVEDV,

left ventricular end-diastolic volume; LVESV, left ventricular end-systolic volume. All values are presented as mean ± SEM.

Table S3. Parameters in *Car3*-deficient (*Car3*$^{-/-}$) mice and their wild-type (WT) littermates at 28 days after MI operation.

/	Sham		MI	
Parameter	WT (n=8)	*Car3*$^{-/-}$ (n=8)	WT (n=8)	*Car3*$^{-/-}$ (n=8)
HR (beats/min)	478.46±19.90	481.86±19.13	473.84±17.22	479.03±18.66
LVIDd (mm)	3.26±0.36	3.60±0.24	4.45±0.17	5.54±0.56
LVIDs (mm)	1.92±0.30	2.12±0.23	3.94±0.23	5.26±0.51
LVEDV (ml)	49.94±5.87	54.70±8.82	91.61±6.39	162.29±23.29
LVESV (ml)	19.56±4.39	20.05±3.15	68.10±9.48	137.41±24.99
LVEF (%)	72.92±5.34	72.81±4.77	24.76±6.89	11.16±3.43
LVFS (%)	41.13±4.47	41.23±3.83	11.44±3.38	5.02±1.60

All values are presented as mean ± SEM.

Table S4. Parameters in *Car3*-deficient (*Car3*$^{-/-}$) mice and their wild-type (WT) littermates pretreated with C646 or DMSO at 7 days after MI operation.

/	Sham	MI		
Parameter	WT +DMSO (n=8)	WT +DMSO (n=8)	*Car3*$^{-/-}$ +DMSO (n=8)	*Car3*$^{-/-}$ +C646 (n=8)
HR (beats/min)	491.47±9.190	491.51±6.39	493.81±9.37	492.53±8.99
LVIDd (mm)	3.26±0.22	3.78±0.40	4.35±0.33	3.87±0.24
LVIDs (mm)	1.90±0.17	2.99±0.28	3.95±0.36	3.35±0.34
LVEDV (ml)	44.85±7.40	68.75±7.38	90.94±11.59	76.38±5.10
LVESV (ml)	16.35±2.73	35.30±5.44	62.66±5.31	45.38±6.01
LVEF (%)	74.56±4.62	40.62±3.22	20.14±5.82	36.58±3.54
LVFS (%)	42.58±4.21	19.42±1.91	11.36±1.72	17.35±1.88

All values are presented as mean ± SEM.

Figure S1

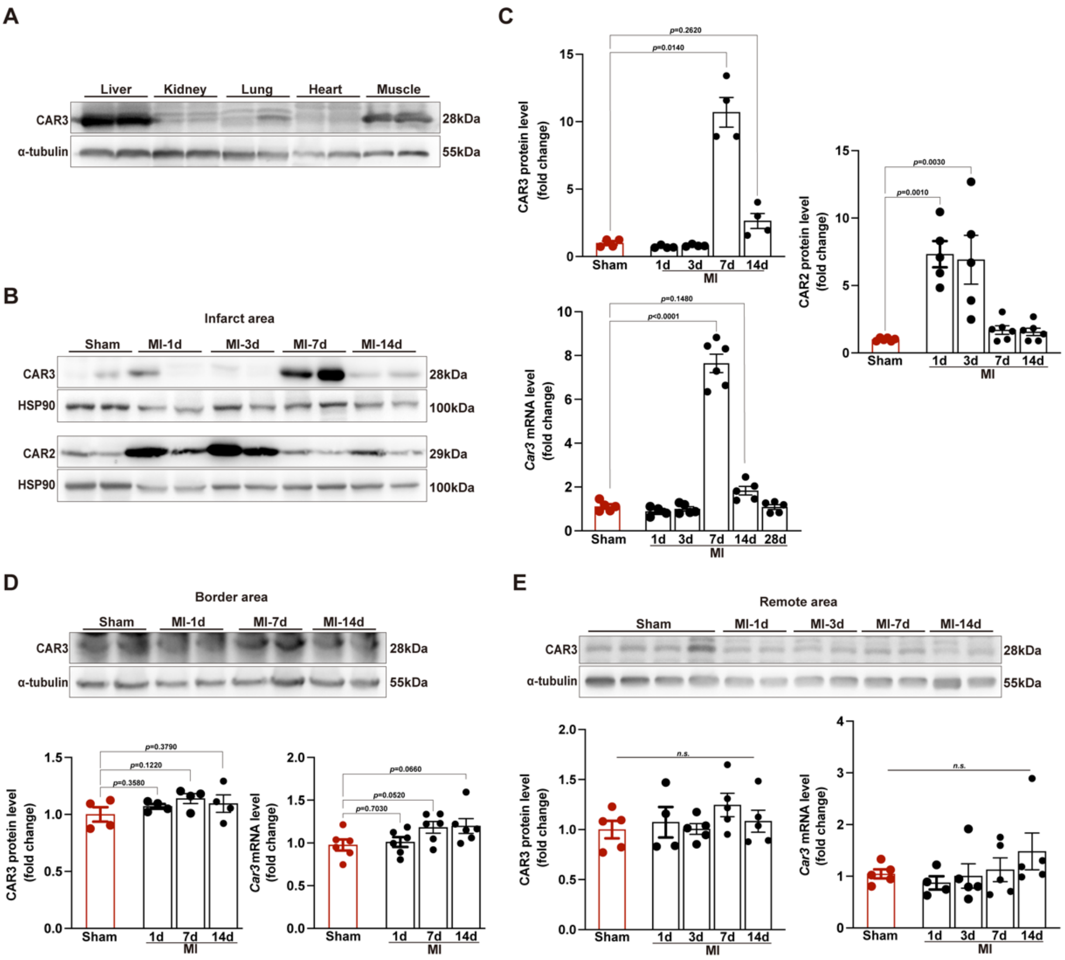
A
Liver
Kidney
Lung
Heart
Muscle
CAR3
α-tubulin
28kDa
55kDa
B
Infarct area
Sham
MI-1d
MI-3d
MI-7d
MI-14d
CAR3
HSP90
CAR2
HSP90
28kDa
100kDa
29kDa
100kDa
C
p=0.2620
p=0.0140
CAR3 protein level (fold change)
Sham
1d
3d
7d
14d
MI
p=0.0030
p=0.0010
CAR2 protein level (fold change)
p=0.1480
p<0.0001
Car3 mRNA level (fold change)
28d
D
Border area
Sham
MI-1d
MI-7d
MI-14d
CAR3
α-tubulin
28kDa
55kDa
p=0.3790
p=0.1220
p=0.3580
p=0.0660
p=0.0520
p=0.7030
E
Remote area
Sham
MI-1d
MI-3d
MI-7d
MI-14d
CAR3
α-tubulin
28kDa
55kDa
n.s.

Figure S2

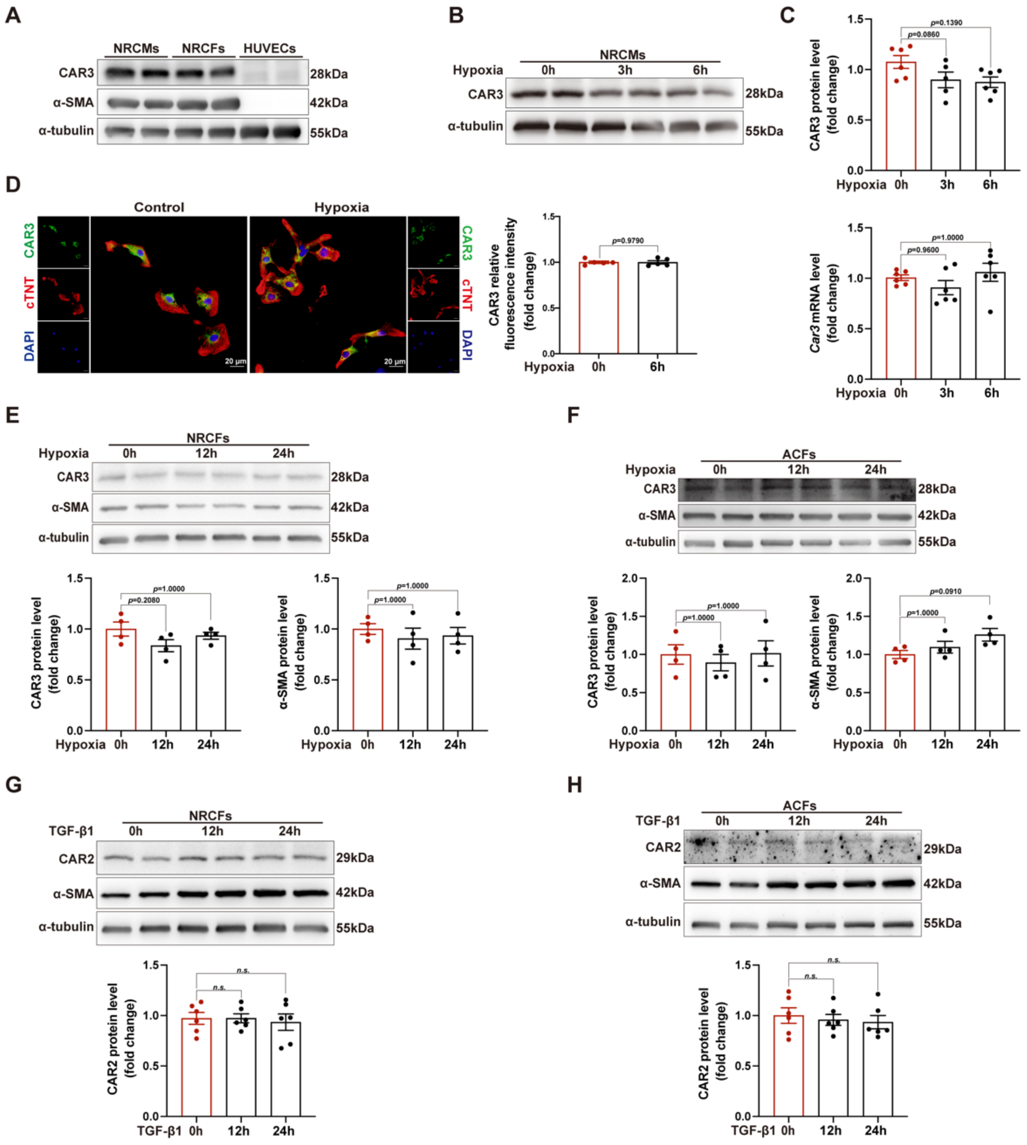

A
NRCMs
NRCFs
HUVECs
CAR3
α-SMA
α-tubulin
28kDa
42kDa
55kDa
B
NRCMs
Hypoxia
0h
3h
6h
CAR3
α-tubulin
28kDa
55kDa
C
CAR3 protein level (fold change)
Car3 mRNA level (fold change)
D
Control
Hypoxia
CAR3
cTNT
DAPI
20 μm
CAR3 relative fluorescence intensity (fold change)
E
NRCFs
F
ACFs
12h
24h
CAR3 protein level (fold change)
α-SMA protein level (fold change)
G
H
TGF-β1
CAR2
29kDa
CAR2 protein level (fold change)
n.s.

Figure S3

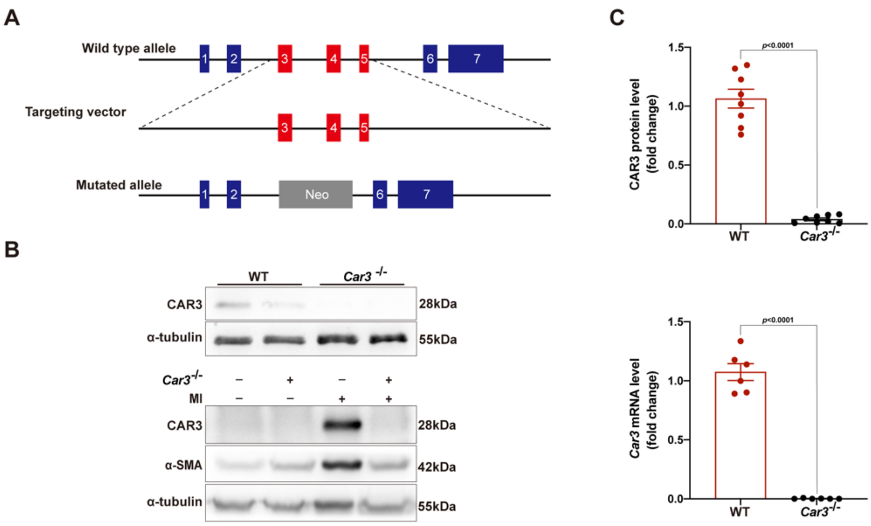

Figure S4

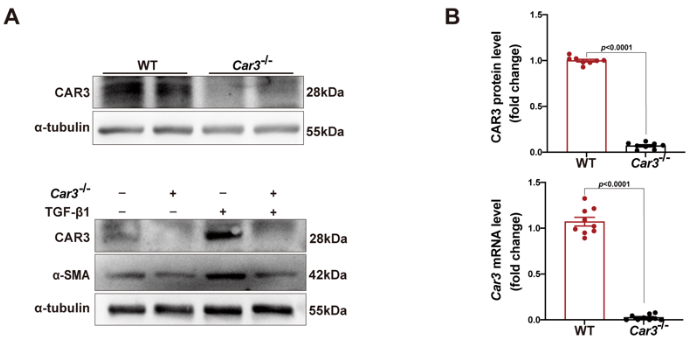

Figure S5

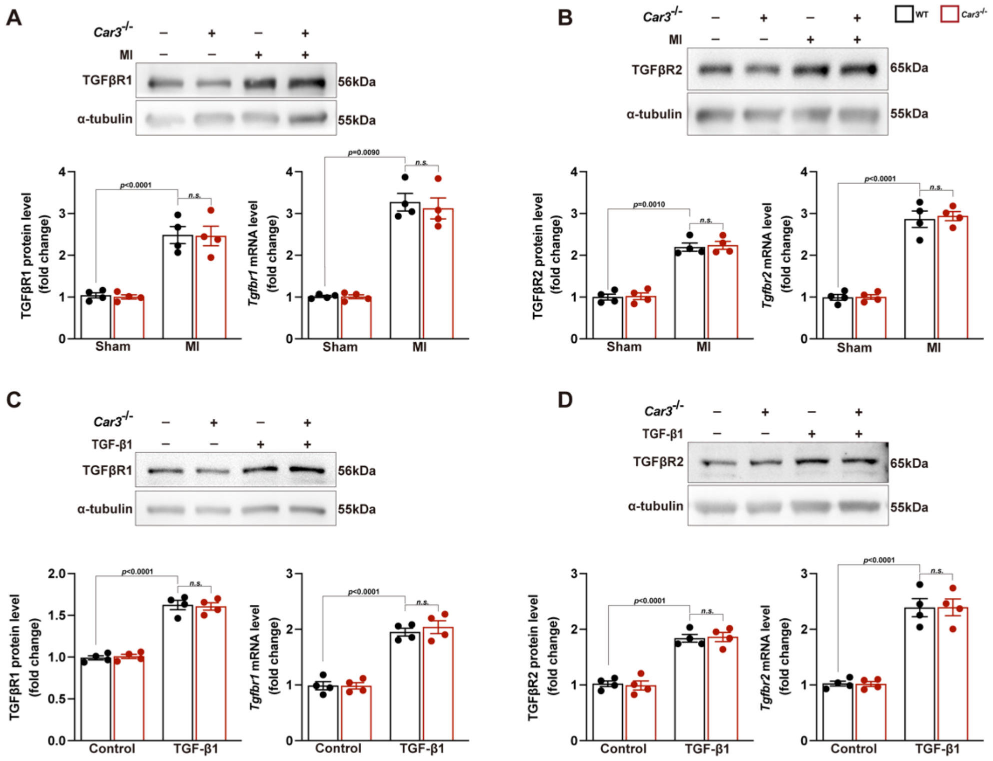

Figure S6

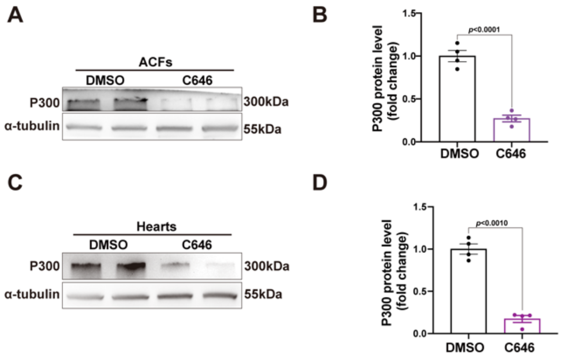

Figure S7

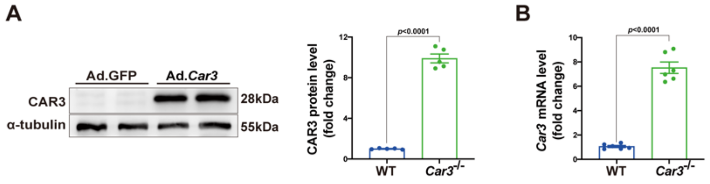

Supplement: Supplementary file 1 — Supplementary materials and methods, figures and tables. [file ijbsv20p1796s1.pdf]
